# Regulation of phosphoinositide metabolism in Apicomplexan parasites

**DOI:** 10.3389/fcell.2023.1163574

**Published:** 2023-09-15

**Authors:** Angela Arabiotorre, Vytas A. Bankaitis, Aby Grabon

**Affiliations:** ^1^ Department of Cell Biology and Genetics, College of Medicine Texas A&M Health Sciences Center College Station, Bryan, TX, United States; ^2^ Department of Biochemistry and Biophysics Texas A&M University College Station, College Station, TX, United States; ^3^ Department of Chemistry Texas A&M University College Station, College Station, TX, United States

**Keywords:** phosphoinositides, eukaryotic parasites, Apicomplexa, lipid signaling, phospholipids

## Abstract

Phosphoinositides are a biologically essential class of phospholipids that contribute to organelle membrane identity, modulate membrane trafficking pathways, and are central components of major signal transduction pathways that operate on the cytosolic face of intracellular membranes in eukaryotes. Apicomplexans (such as *Toxoplasma gondii* and *Plasmodium* spp.) are obligate intracellular parasites that are important causative agents of disease in animals and humans. Recent advances in molecular and cell biology of Apicomplexan parasites reveal important roles for phosphoinositide signaling in key aspects of parasitosis. These include invasion of host cells, intracellular survival and replication, egress from host cells, and extracellular motility. As Apicomplexans have adapted to the organization of essential signaling pathways to accommodate their complex parasitic lifestyle, these organisms offer experimentally tractable systems for studying the evolution, conservation, and repurposing of phosphoinositide signaling. In this review, we describe the regulatory mechanisms that control the spatial and temporal regulation of phosphoinositides in the Apicomplexan parasites *Plasmodium* and *T. gondii*. We further discuss the similarities and differences presented by Apicomplexan phosphoinositide signaling relative to how these pathways are regulated in other eukaryotic organisms.

## Introduction

### Phosphoinositide signaling in eukaryotes

**FIGURE 1:**
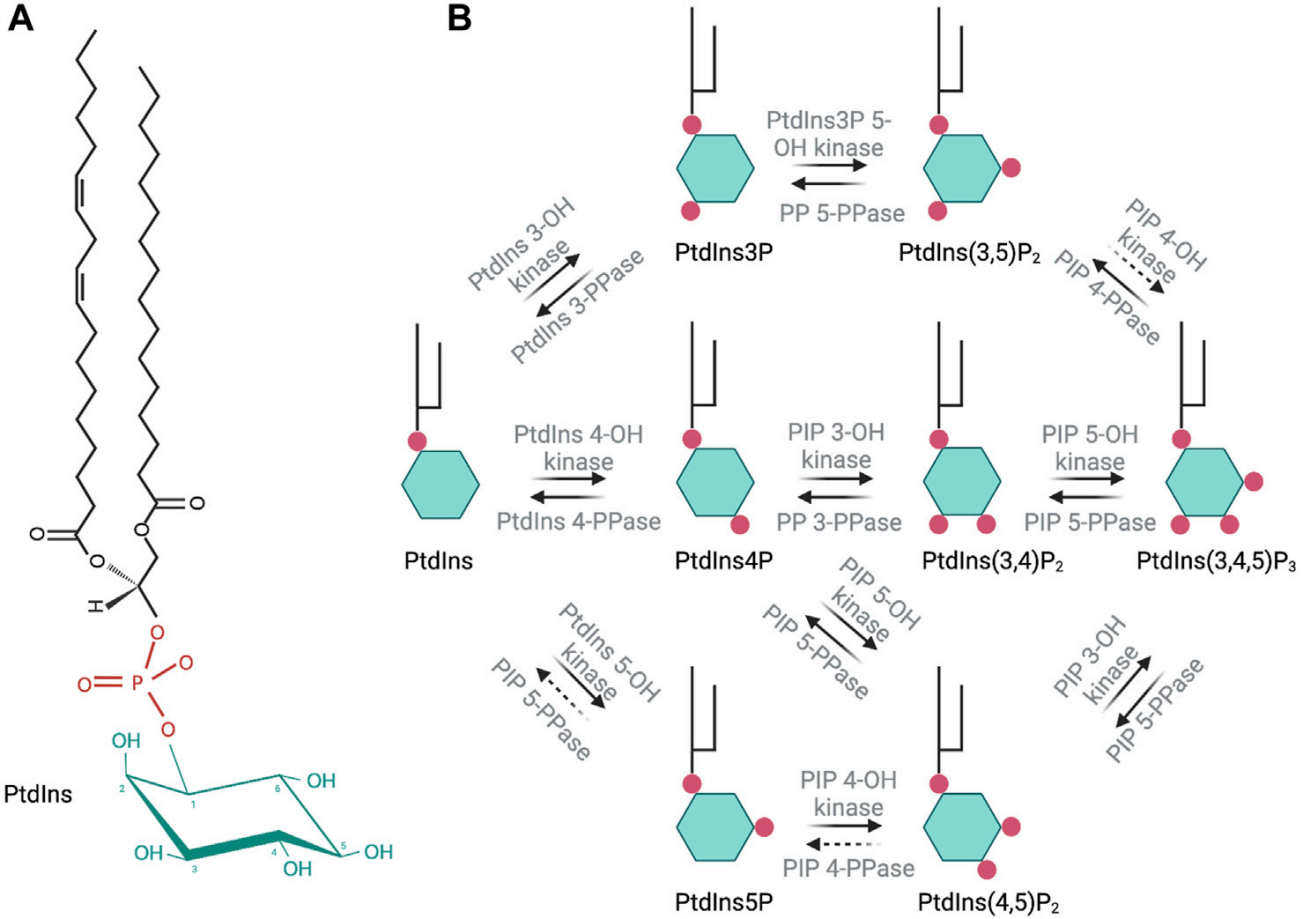
Molecular structure of phosphatidylinositol and schematic representation of the seven phosphoinositide species and their synthesis in eukaryotic cells. Enzymatic activity involved in the synthesis or degradation of these species is indicated over the respective arrows. Dashed arrows indicate an enzymatic activity that has not been fully described.

Phosphoinositides (PIPs) contribute to organelle identity and regulate both membrane trafficking and other signal transduction events at eukaryotic cellular membranes (Figure 1). PIPs are produced by phosphorylation of the inositol head group of phosphatidylinositol (PtdIns). Diversity of PIP isoforms is greater in higher eukaryotes. For instance, yeast produces five distinct PIP isoforms, namely, PtdIns3P, PtdIns4P, PtdIns5P, PtdIns(3,5)P_2_, and PtdIns(4,5)P_2_, whereas metazoan organisms generally produce seven isoforms: those found in yeast plus PtdIns(3,4)P_2_ and PtdIns(3,4,5)P_3_. Each PIP isoform executes its own unique biological activities, and on this basis, these molecules are considered to create a signaling “code.” This PIP code is the foundation of a major intracellular signaling system that interfaces with virtually all pathways that control and regulate cell growth, motility, division, and death in eukaryotic cells.

The PIP code is read by specific PIP-binding domains of effector proteins. The first such domain described was the Pleckstrin homology (PH) domain from phospholipase C delta (PLCδ), which is a high-affinity PtdIns(4,5)P_2_-binding module (Garcia et al., 1995; Lemmon et al., 1995). This discovery paved the way for the identification of many other PIP-binding modules (e.g., FERM, FYVE, PX, ENTH/ANTH, and PROPPIN domains) that exhibit varying degrees of PIP-binding specificity and affinity (Hammond and Balla, 2015). This chemical diversity allows intricate and combinatorial fine-tuning of PIP-dependent regulation of complex cell functions. In that regard, PIPs are uniquely eukaryotic phospholipids.

PIPs are not homogeneously distributed along the surface of intracellular membranes. Rather, these are organized into dynamic domains that impose spatial and temporal regulation of peripheral membrane protein binding and/or enzymatic reactions. Thus, PIP spatiotemporal location in a cell is tightly controlled. Cellular PIP levels are regulated through the activity of phosphatidylinositol kinases (PIKs), phosphoinositide kinases (PIPK), and PIP phosphatases (Figure 1). PIKs catalyze the phosphorylation of PtdIns on the inositol ring at positions 3′, 4′ or 5′-OH. PIPK phosphorylate the inositol ring of PIPs at the 3′, 4′ or 5′-OH positions. Phosphatases catalyze the removal of a -PO_4_ group at 3′, 4′ or 5′-OH. Dysregulation of PIP homeostasis in human cells is strongly associated with cancer, primary immunodeficiencies, developmental disorders, and various other pathologies (Di Paolo and De Camilli, 2006; Balla, 2013).

Organisms of the phylum Apicomplexa are complex, single-celled eukaryotes that exhibit a sophisticated parasitic lifestyle. Recent advances in cellular and molecular studies of Apicomplexans demonstrate the role of phosphoinositide signaling networks at many key points of parasite biology. The unique biological niche occupied by Apicomplexa, and the resulting adaptations that come with it, identifies these parasites as exceptional models for the study of novel and ancient aspects of phosphoinositide biology. In this review, we consider how phosphoinositide signaling is regulated in Apicomplexa, and how this information might be used to better understand both parasite biology and eukaryotic lipid signaling.

### Apicomplexan parasites

Members of the phylum Apicomplexa are obligate intracellular parasites that infect both vertebrate and invertebrate hosts and are significant disease-causing agents of both animals and humans. There are seven genera that infect humans. These include i) *Plasmodium* spp., which are the causative agents of malaria, ii) the opportunistic human pathogen *Toxoplasma gondii*, iii) the malaria-like parasite *Babesia* spp., iv) the animal parasite and opportunistic human pathogen *Cryptosporidium* spp., and v) the human intestinal disease parasites *Isospora belli*, *Cyclospora cayetanensis*, and *Sarcocystis* spp. (Ackers, 1997; Chakraborty et al., 2017). Among the Apicomplexan parasites, *Plasmodium* and *T. gondii* are the most experimentally tractable as these organisms can be cultured *in vitro*, can be studied in rodent models of infection, and are amenable to genetic manipulation. Thus, these two organisms represent the focus of this review.

Because the Apicomplexan organisms are obligate intracellular parasites, these organisms must invade their respective host cells and establish a suitable intracellular environment that supports their replication and proliferation. In that regard, most Apicomplexans produce an elaborate intracellular compartment termed the parasitophorous vacuole (PV) through which the parasites scavenge nutrients from the host to power parasite cell division or differentiation. In the acute phases of infection, the parasites escape the PV and egress from the host cell in order to propagate the infection throughout the tissue. Apicomplexans have complex developmental cycles which include both acute and latent phases, and these organisms are able to pass through a variety of insect vectors and infect a variety of animal hosts.

### The unique Apicomplexan endomembrane system

Apicomplexans possess highly specialized endomembrane structures that allow these parasites to execute their complex life cycles. As members of the superphylum Alveolata, Apicomplexans have evolved a network of flattened vesicles located below the plasma membrane called the inner membrane complex (IMC). The IMC spans the periphery of the cell, except at the basal and apical poles and at the micropore/cytostome (an invagination or cup-shaped structure at the plasma membrane). The IMC is involved in calcium storage, regulation of cell division, and regulation of gliding motility (Harding and Meissner, 2014). The apicoplast is another unique endomembrane compartment, a non-photosynthetic plastid-like organelle of red algal origin that houses anabolic activities indispensable for parasite growth and viability. These activities include synthesis of heme and fatty acids via the FASII complex and production of isoprenoid compounds (Coppens et al., 2014; Bergmann et al., 2020).

Apicomplexans derive their name from a signature intracellular structure termed the ‘apical complex,’ a collection of specialized secretory organelles unique to the phylum (i.e., micronemes, rhoptries, and dense granules) along with the apicoplast (Gubbels and Duraisingh, 2012). In some Apicomplexans, the apical complex also includes an anterior-most conoid that lies within a microtubule cylinder called the polar ring. Micronemes, the smallest secretory organelles, are required for parasite egress from host cells. Rhoptries are larger compartments that exhibit club-shaped morphologies with the slim end attached to the apical pole of the parasite. Release of rhoptry and microneme contents is required for formation of the moving junction, that is, a ring-like protein structure that moves from the apical to the posterior end of the parasite to carry out the formation of the PV membrane (PVM) during host cell invasion. The primary function of the PVM is to envelop the intracellular parasites and sequester these from the host cytoplasm to avoid interface with the host endocytic pathway. This is facilitated by secretion of factors from dense granules (DGs), secretory organelles distributed throughout the parasite cytoplasm. DG cargos are released following host invasion and during the intracellular life of the parasite and include factors involved in nutrient acquisition from the host, manipulation of host cell signaling pathways, and maintenance of the PVM (Gubbels and Duraisingh, 2012; Bai et al., 2018).

### Apicomplexan life cycles

**FIGURE 2:**
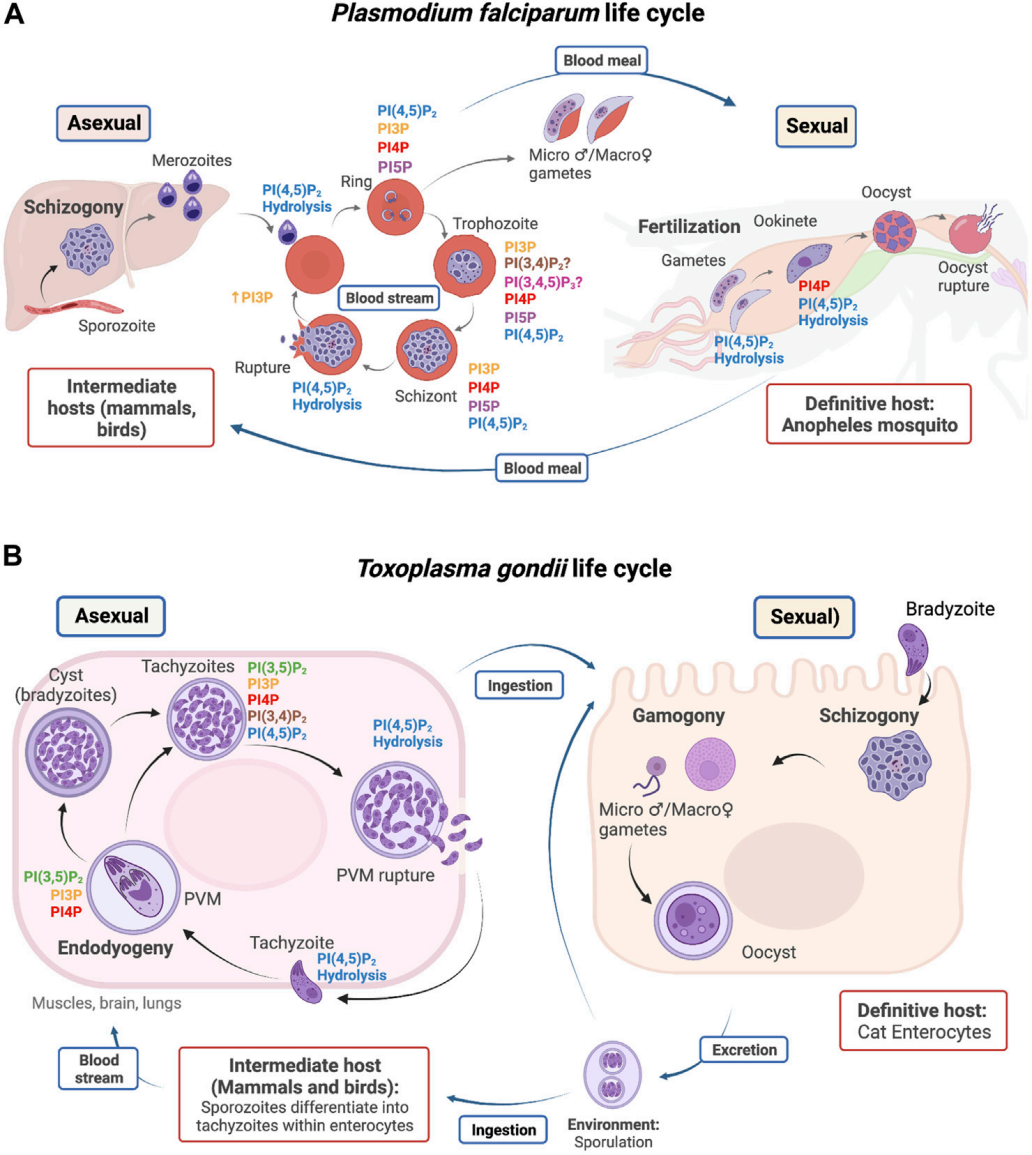
Life cycle of *P. falciparum* **(A)** and *T. gondii* **(B)**. PIP detection and utilization is highlighted in specific stages during the parasite life cycles. Host types are outlined in red, and reproductive stages are outlined in blue.

Members of the phylum Apicomplexa exhibit a wide range of morphologies, habitats, cell division processes, and life cycles. These subjects are only briefly treated here as recent reviews discuss them in detail (Nilsson et al., 2015; Venugopal et al., 2020b; Martorelli Di Genova and Knoll, 2020). *Plasmodium* parasites are transmitted to humans by the bite of an infected mosquito, which injects sporozoites into the blood stream. The parasites then reach the liver and develop into merozoites through schizogony or asexual replication (Figure 2A). Merozoites escape the liver and enter the blood stream to infect erythrocytes. Intraerythrocytic merozoites adopt a ring-like morphology and undergo further differentiation to trophozoites, which divide by schizogony (schizont stage) to produce several merozoites. Erythrocyte lysis leads to liberation of merozoites and to repeated infection cycles of other erythrocytes. These cycles are responsible for the severe symptoms that accompany malarial disease. Some parasites differentiate into micro and macrogametocytes–the sexual erythrocytic stages. When ingested by *Anopheles* mosquitoes during a blood meal, the macrogametocyte is fertilized by a microgametocyte in the mosquito’s stomach. The zygote develops into a motile and elongated ookinete that invades the midgut wall and develops into an oocyst to produce sporozoites in the salivary glands of the mosquito (Read et al., 1993; Francia & Striepen, 2014).

*T. gondii* infects the intestinal track of its definitive host, the domestic cat (Figure 2B). Cats become infected directly by ingestion of sporulated oocysts, at which point either sexual reproduction in the intestine or asexual multiplication in peripheral organs can take place (Ferguson et al., 1974; Francia & Striepen, 2014). The feces of infected cats contain oocysts that sporulate in the environment and are accidentally ingested by humans and other vertebrates (i.e., the intermediate hosts). Shortly after ingestion, oocysts differentiate into tachyzoites and invade neural and muscle tissue, where they divide asexually through internal budding (referred to as endodyogeny) and develop into cyst bradyzoites. When the host becomes immunocompromised, bradyzoites differentiate back into tachyzoites and cycle through rounds of replication until the PVM is disrupted via the activity of a *T. gondii* perforin-like protein. Host cell membranes burst rapidly thereafter, and the lytic cycle starts again when a tachyzoite invades and reproduces in a new host cell, thereby propagating an acute infection of the affected tissue (Roiko and Carruthers, 2013).

### Phosphoinositide signaling in Apicomplexa

Apicomplexans possess the neutral and polar lipid species generally found in eukaryotic cells but produce unique lipids as well. Analyses of genes encoding metabolic enzymes involved in lipid production in Apicomplexa, when coupled with lipid labeling and deprivation experiments, demonstrate that these parasites encode the enzymatic machinery to synthesize many lipid species. However, some essential lipids must be scavenged from the host cell. One such example is salvaging of host cholesterol by *T. gondii,* an essential activity because *T. gondii* do not express the enzymes required for sterol production (Coppens et al., 2000; Coppens et al., 2014; O’Neal et al., 2020). The phospholipid profiles of *T. gondii* and *Plasmodium* are also maintained through a balance of endogenous synthesis and scavenge mechanisms. PtdIns represents a major class of membrane phospholipids in *T. gondii* and *Plasmodium*. It constitutes ∼7% of the total phospholipid content, and this value is similar to that found in mammalian cells (Vial et al., 2003; Welti et al., 2007). PtdIns metabolism has not been studied in detail in Apicomplexan organisms. The genomes of *T. gondii*, *Plasmodium*, and *Eimeria* all encode a PtdIns synthase (PIS) (Séron et al., 2000; Wengelnik and Vial, 2007; Kong et al., 2018). The *T. gondii* PIS (TgPIS) is an essential enzyme that localizes to the Golgi network and consumes CDP-diacylglycerol (CDP-DAG) and myoinositol as precursors in *de novo* PtdIns synthesis. The parasite is also capable of salvaging longer-chain PtdIns molecular species (C38/C40) from the host cell (Ren et al., 2020).

The current cohort of Apicomplexan PIPs has been identified and localized by exploiting protein domains with high affinities and specificities for individual PIP species (PIP biosensors) and through parasite lipid profiling. The function of these PIPs has also been studied using controlled expression strategies targeting PtdIns and PIP kinases responsible for the synthesis of individual PIP species. As is the case in all other eukaryotes studied thus far, Apicomplexan PIPs are essential molecules for mediating important cell functions, including the virulence of these organisms. For instance, PIPs are involved in apicoplast homeostasis, in host cell invasion by *T. gondii* (Daher et al., 2015; Bullen et al., 2016), in the transport and fusion of endosomes with lysosome-like compartments, and in motility and host cell egress processes in *Plasmodium* (Vaid et al., 2010; Brochet et al., 2014).

Understanding how PIP pools are formed and maintained in eukaryotes remains an intense area of study in contemporary cell biology. Although much effort is invested in studying the enzymes that produce and consume PIPs, fundamental aspects of how PIP production is regulated and physically organized are still not completely understood. Moreover, most of the information on the metabolism and physiological roles of inositol lipids is derived from studies in a limited set of model organisms. Emerging evidence suggests that Apicomplexa evolved from the secondary endosymbiosis of a photosynthetic alga by an ancestral eukaryotic cell (McFadden, 2000; Oborník, 2020). As such, these organisms provide an opportunity to study the physiological integration of complex PIP signaling systems in unicellular life forms with unique evolutionary trajectories. In the case of Apicomplexa, these trajectories outline transitions from what might have been free-living phototrophs to obligate non-photosynthetic animal parasites. Additionally, PIP signaling mechanisms in Apicomplexa offer intriguing possibilities for development of potential drug targets to combat parasitic diseases.

Recently, two reviews focused on the PIPs in Apicomplexa have been published. The first covers PIP distribution and cellular functions and studies of PtdIns and PIP-kinases in *T. gondii* and *Plasmodium* (Wengelnik et al., 2018). The second focuses on PIP-binding proteins involved in endocytic trafficking and autophagy in these parasites (Cernikova et al., 2019). The focus of this review is to address regulatory mechanisms that control the spatial and temporal regulation of PIPs in *Plasmodium* and *T. gondii* and to draw comparisons and distinctions of these mechanisms relative to those described for other eukaryotic systems, particularly mammalian cells and the model organism *S. cerevisiae*, which we refer to as “yeast” in this review.

## PtdIns3P and its higher-order derivatives

### The Apicomplexan PtdIns3P kinases

**FIGURE 3:**
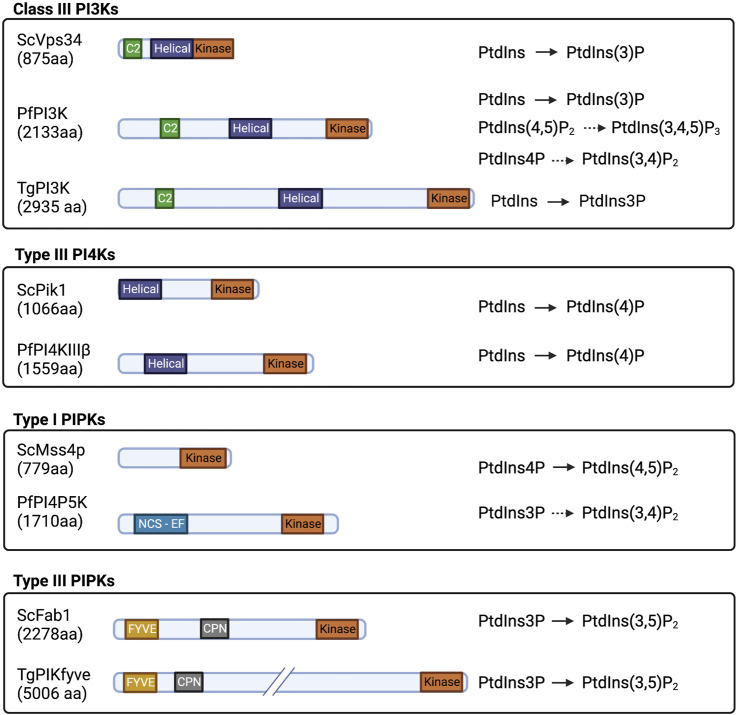
Domain organization and substrate specificity of current known Apicomplexan PIKs and PIPKs and their yeast homologs drawn to scale. Kinase: PI/PIP kinase domain; Helical: helical domain; C2: C2 domain; FYVE: FYVE zinc finger domain; CPN: Chaperonin; and NCS-EF: NCS-like domain with EF hands.

**FIGURE 4:**
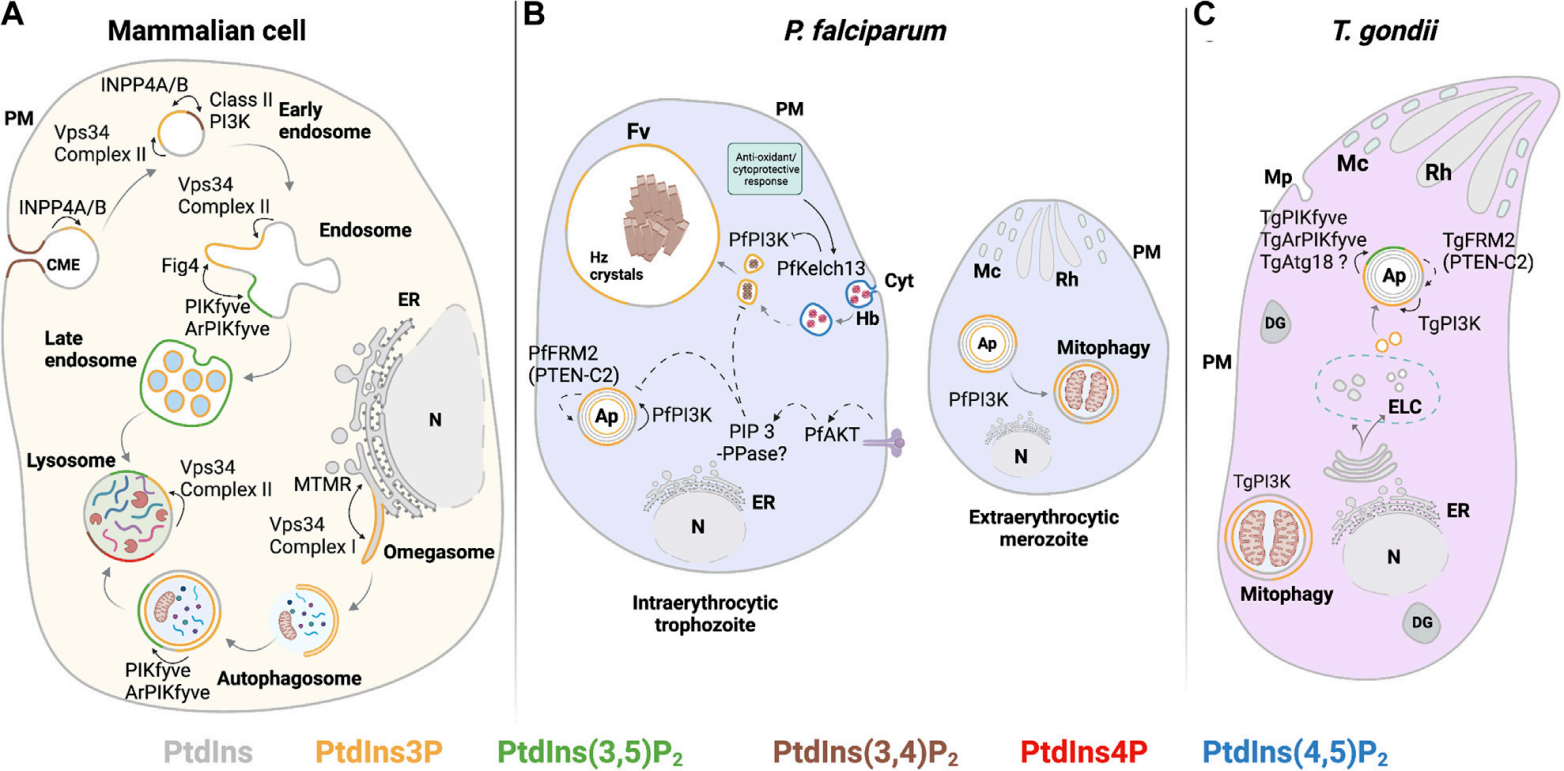
PIPs involved in the endocytic system and autophagy. **(A)** Mammalian: in preformed CME vesicles PtdIns(3,4)P_2_ is dephosphorylated by 4-phosphatases (INPP4A/B) to form PtdIns3P. In early endosomes, PtdIns3P is also generated from PtdIns by the class III PI3K Vps34 complex II. Maturation to late endosomes involves PtdIns(3,5)P_2_ synthesis. PtdIns3P synthesis by class III PI3K Vps34 complex II is required for initiating autophagy at the omegasome. Autophagosome maturation and fusion with the lysosome depend on conversion of PtdIns3P to PtdIns(3,5)P_2_ by PIKfyve kinase. **(B)** *P. falciparum*: PtdIns(4,5)P_2_ is detected in the cytostome and cytostomal vesicles. Maturation of cytostomal vesicles is accompanied by reduction of their size and enrichment of PtdIns3P by PfPI3K activity. PtdIns3P is also observed in the food vacuole membrane, autophagosomes, and at the apicoplast. Negative regulation of PtdIns3P levels in these compartments might include the PTEN C2-like domain in PfFRM2, the activation of PfAKT signaling, and PfKelch13-dependent PfPI3K proteolysis. At the extracellular merozoite stage, PfPI3K activity regulates autophagy-related processes such as mitophagy. **(C)** *T. gondii*: PtdIns3P is produced by TgPI3K at the outer and inner membranes of the apicoplast and at neighboring vesicles. PtdIns(3,5)P_2_ production from PtdIns3P by the complex TgPIKfyve, TgArPIKyve, and possibly TgAtg18 is key to apicoplast maintenance. TgPI3K activity is also involved in mitophagy during parasite starvation. The PTEN-C2 domain of TgFRM2 may negatively regulate PtdIns3P at the apicoplast. *Solid line:* proposed reaction in previous publications and *dashed line:* proposed reaction in this review. Abbreviations in Figures 4–6: PM: plasma membrane; ER: endoplasmic reticulum; N: nucleus; Cyt: cytostome; Mp: micropore; Mc: microneme; Rh: Rhotry; Fv: food vacuole; Hz crystals: hemozoin crystals; Ap: apicoplast; DG: dense granule; ELC: endocytic-like compartment.

Phosphatidylinositol-3-phosphate (PtdIns3P) is produced by phosphorylation of the PtdIns inositol head group on the 3′-OH position. This reaction is catalyzed by class II and class III PtdIns 3-OH kinases (PI3Ks) in mammals (PIK3C2 and PIK3C3). Unicellular organisms express one PI3K, the class III Vps34 (Vanhaesebroeck et al., 2010; Brown and Auger, 2011). Both *Plasmodium* and *T. gondii* encode a single PI3K Class III/Vps34 enzyme that is responsible for cellular all PtdIns3P synthesis: PfPI3K (PF3D7_0515300) and TgPI3K (TGME49_215700). These enzymes localize primarily to apicoplast and vacuolar and vesicular compartments, in both parasites where pools of PtdIns3P are produced (Figures 4B,C). Both kinases exhibit a conserved PI3K Class III domain organization consisting of i) an N-terminal C2 domain, ii) a PIK domain (PI3K family accessory domain that has been suggested to be involved in substrate presentation), and iii) a C-terminal 3-OH kinase catalytic domain. Both PfPI3K and TgPI3K are essential for parasite survival (Vaid et al., 2010; Brown and Auger, 2011; Daher et al., 2015), and both enzymes are predicted to be substantially larger than any other PI3K described thus far (Figure 3).

PI3K class III/Vps34 is found in two principal complexes in mammalian cells, namely, complex I and complex II (Fan et al., 2011; Rostislavleva et al., 2015). Complex I is composed of Vps34, Vps15, Beclin 1 (Vps30), and an autophagy-related gene 14L (ATG14L), and this complex is responsible for generating PtdIns3P in the endoplasmic reticulum (ER). It is this pool that drives autophagosome preassembly machinery at the omegasome (Mizushima et al., 2008). In complex II, ATG14L is replaced by UVRAG (Vps38 in yeast), which facilitates targeting of the complex to early endosomes to produce PtdIns3P. Recruitment of complex II occurs in an Rab5-dependent manner (Figure 4A). This process potentiates maturation of early Rab5-positive vesicles to late Rab7-positive vesicles for further fusion with lysosomes (Shin et al., 2005; Vanhaesebroeck et al., 2010; Burke, 2018). Of these regulatory factors, only a candidate Vps15 ortholog is annotated by both *T. gondii* and *Plasmodium* genomes, and a prospective Vps30 is encoded by *T. gondii* (Supplementary Table S1) (PlasmoDB (v56); ToxoDB (v56)). The dedicated components that define complexes I and II (ATG14L and UVRAG, respectively) and aid in target membrane recognition have no obvious counterparts in these parasites (Besteiro, 2017). *Plasmodium* and *T. gondii* may express novel and specialized accessory proteins adapted for targeting Apicomplexan Vps34 enzymes to their unique endomembranous compartments.

### PtdIns3P in starvation and autophagy during the Apicomplexan life cycle

Among the intraerythrocytic stages of *P. falciparum*, the highest levels of PtdIns3P are detected during transition from the ring to the schizont phase. The PfPI3K is localized to the limiting membranes of vesicular compartments and the food vacuole (Vaid et al., 2010). The food vacuole is the organelle where hemoglobin (Hb) is digested, and Hb represents the primary amino acid nutrient source for the parasite. Phosphoproteome profiling experiments reveal that PtdIns signaling and PfPI3K activity are elevated in extraerythrocytic merozoites relative to the schizont stage (Figure 2A). Moreover, ubiquitination and autophagy pathways are also upregulated in this stage (Lasonder et al., 2015). Upregulation of ubiquitination and autophagy is consistent with the observation that extraerythrocytic merozoites lack the food vacuole (Dluzewski et al., 2008), thereby forcing the parasite to undergo autophagic digestion of organelles no longer required by extraerythrocytic merozoites as a mechanism to combat starvation. Such observations reveal a potential role for PfPI3K in the regulation of starvation-induced processes during the extraerythrocytic stages (Lasonder et al., 2015) (Figure 4B). Similarly, the *T. gondii* TgPI3K activity stimulates mitochondrial fragmentation during mitophagy when the parasite encounters starvation conditions (Figure 4C) (Ghosh et al., 2012).

The role of autophagy as a life-sustaining process under the conditions of starvation stress is well-established (Sinai and Roepe, 2012; Qi et al., 2018). Elucidating the regulatory mechanisms that govern PtdIns3P synthesis is crucial for understanding how energy balance and cell homeostasis are maintained under starvation conditions in protozoan organisms. In that regard, genome-wide association studies report that PfPI3K/PtdIns3P levels are modulated by the action of an AKT ortholog (PfAKT) in *P. falciparum* as elevated PfAKT expression in transgenic parasites induces a ∼2-fold increase in PtdIns3P levels (Mbengue et al., 2015). Akt is a master regulator of diverse cellular functions in mammals that include survival, growth, metabolism, migration, and differentiation (Vivanco and Sawyers, 2002; Sugiyama et al., 2019). In mammals, Akt activation can lead to diminished PtdIns3P production. For instance, activation of mTOR1 by the PI3K class I/Akt axis inactivates Vps34-containing complexes and suppresses autophagy (Marat and Haucke, 2016). A reaction where Akt stimulation increases PtdIns3P levels has not been well-described in other organisms. One intriguing possibility is that the absence of the canonical components of Vps34 complexes in Apicomplexans generates an outcome for Akt-dependent PtdIns3P signaling that diverges from those classically observed in mammalian cells.

Downregulation of PtdIns3P signaling is executed by myotubularin 1 (MTM1) and MTM-related proteins that dephosphorylate PtdIns3P to PtdIns. MTM genes are nearly ubiquitously distributed throughout Eukaryota (Laporte et al., 1996; Vergne and Deretic, 2010). Thus far, *Cryptosporidium parvum* and *C. hominis* are the only Apicomplexans that encode an MTM-like protein. *Plasmodium* and *T. gondii* lack obvious MTM orthologs (Supplementary Table S1) (Abrahamsen et al., 2004; Kerk and Moorhead, 2010; Laporte et al., 2003). Whether this activity is missing in *Plasmodium* and *T. gondii*, or whether it is fulfilled by a protein with no obvious homology to the myotubularins, remains to be determined.

### PtdIns(3,5)P_2_ synthesis in Apicomplexa

The basic mechanisms underlying the maintenance of PtdIns(3,5)P_2_ levels are highly conserved. PtdIns3P 5-kinase (Fab1 in yeast and PIKfyve in mammals) phosphorylates PtdIns3P on the inositol ring at the 5′-OH position to produce PtdIns(3,5)P_2_. PIKfyve and Fab1 both reside in large protein complexes that tightly regulate PtdIns(3,5)P_2_ production. This complex comprises four more proteins in yeast: Figure 4, Vac14, Vac7, and Atg18. PtdIns(3,5)P_2_ turnover is proposed to be mainly catalyzed by Figure 4, a 3,5-bisphosphate 5-phosphatase (Sac3 in mammals), which both positively and negatively regulate PtdIns(3,5)P_2_ production. Vac14 (ArPIKfyve in mammals) functions as a scaffold protein that promotes PtdIns(3,5)P_2_ synthesis. Vac7 is an essential protein in yeast, but its function in Fab1 activation is not clear. Vac7 metazoan homologs have not been detected. Finally, Atg18 negatively regulates PtdIns(3,5)P_2_ production, likely through its interaction with Vac14. In yeast, all of these factors localize to the vacuolar membrane, whereas mammalian PIKfyve and ArPIKfyve localize to membranes of the endocytic system (Figure 4A) (Gary et al., 2002; Botelho et al., 2008; Jin et al., 2008; Shisheva, 2008; Hasegawa et al., 2017).

PtdIns(3,5)P_2_ in *T. gondii* is localized to a population of uncharacterized vesicles distributed throughout the cytoplasm of tachyzoites (Figure 4C), and its synthesis is associated with apicoplast biogenesis/inheritance (Daher et al., 2015; Daher et al., 2016). PtdIns(3,5)P_2_ has not yet been reported in *Plasmodium*. Whether this reflects a genuine absence of PtdIns(3,5)P_2_ in this parasite, or very low levels that fall below available thresholds of detection, is not clear. Putative homologs of Fab1/PIKfyve, Vac14, and Atg18 have been described and are essential for parasite growth in *T. gondii* (Daher et al., 2015; Bansal et al., 2017). Depletion of TgPIKfyve (TGME49_256920), TgArPIKfyve (TGME49_244040), or TgAtg18 results in similar apicoplast biogenesis/inheritance disruption phenotypes as those previously observed in strains where PtdIns3P signaling is blocked (Tawk et al., 2011). In independent studies, colocalization of TgPIKfyve and TgAtg18 with the otherwise uncharacterized PtdIns(3,5)P_2_-containing vesicular structures was also observed (Daher et al., 2015; Bansal et al., 2017). These data suggest *T. gondii* employs a conserved PtdIns(3,5)P_2_ regulatory complex to control vesicle trafficking to the apicoplast (Figure 4C).

The only component of the PtdIns(3,5)P_2_ regulatory complex described thus far in *Plasmodium* is the Atg18 ortholog PfAtg18 (Bansal et al., 2017; Sudhakar et al., 2021). PfAtg18 and TgAtg18 bind PtdIns3P *in vitro*. TgAtg18 also binds PtdIns(3,5)P_2_, but PfAtg18 does not. While these data are consistent with the notion that PtdIns(3,5)P_2_ is absent in *Plasmodium*, candidates for a PIKfyve lacking the FYVE finger domain and ArPIKfyve and Figure 4 homologs are listed in the *Plasmodium* database (PlasmoDB v56) (Supplementary Table S1). Prospective *P. berghei* PIKfyve- and ArPIKfyve-like proteins score as essential activities in an *in vivo* screen that monitors growth rate phenotypes of strains individually deficient in each of 2,578 *P. berghei* genes (Bushell et al., 2017). The available data suggest that *Plasmodium* PIKfyve is not involved in PtdIns3P binding and its conversion to PtdIns(3,5)P_2_. Further characterization of these protein candidates, along with implementation of more sensitive PtdIns(3,5)P_2_ detection methods, is required to determine whether or not *Plasmodium* produce PtdIns(3,5)P_2_.

### PtdIns3P and PtdIns(3,5)P_2_ in Apicomplexa are not restricted to endosome trafficking pathways

In mammalian and yeast cells, PtdIns3P localizes primarily to membranes of early endosomes, multivesicular bodies (MVBs), and MVB transport intermediates, where it regulates membrane receptor sorting and intralumenal vesicle formation (Gillooly et al., 2000; Petiot et al., 2003). Vsp34 complex II is responsible for producing PtdIns3P in these compartments, and PtdIns(3,5)P_2_ is the signature marker for late endolysosomal membranes in higher eukaryotes and for yeast vacuoles (Figure 4A). PtdIns(3,5)P_2_ synthesis is associated with endomembrane homeostasis via regulation of homotypic and heterotypic fusion and fission events involving early and late endosomes and MVB membranes (Ikonomov et al., 2006; Nicot et al., 2006; Whitley et al., 2009; Duex et al., 2006).

Unlike in most eukaryotic cells, endocytosis and exocytosis in Apicomplexan parasites take place within the context of a host eukaryotic cell. Hence, specialized endocytic structures (micropore/cytostome) and secretory organelles (rhoptries, micronemes, and dense granules) are employed to carry out these processes. For instance, endocytosis of Hb by intraerythrocytic *Plasmodium* parasites takes place at the cytostome. When the cytostome matures and pinches off, the resulting endosomal structures containing erythrocytic material are trafficked to the food vacuole where Hb is metabolized to the non-toxic product hemozoin (Lazarus et al., 2008; Milani et al., 2015). PtdIns3P is detected in the subsets of these erythrocytic material-containing endosomal structures in the cytoplasm and in the food vacuole membrane (Figure 4B) (Tawk et al., 2010). Moreover, PtdIns3P synthesis is linked to the transport of Hb-loaded vesicles to the food vacuole for degradation (Vaid et al., 2010).

PtdIns3P levels in *P. falciparum* are regulated via proteolytic degradation of PfPI3K by PfKelch13, an adapter for E3 ubiquitin ligases involved in cytostome biogenesis and/or function (Mbengue et al., 2015; Yang et al., 2019). PfKelch13 shows homology to the mammalian kelch-like ECH-associated protein 1 (Keap1) which controls adaptive response to oxidative stress. A mutation in the PfKelch13 propeller domain affects substrate affinity with a consequent decrease in PfPI3K proteolysis and elevation in bulk intracellular PtdIns3P (Ariey et al., 2014; Mbengue et al., 2015). PfKelch13 localizes to the ER, to Rab5A/5B/6/7/11A-positive vesicles, and to structures adjacent to the cytostome. Co-immunoprecipitation analyses show PfKelch13 physically associates with multiple factors that regulate vesicle trafficking and endocytosis in eukaryotes, i.e., members of the Rab GTPase family (Sar1, Sec23, and others), but not with PfPI3K (Gnädig et al., 2020). The precise function of PfKelch13 remains to be determined. The structural similarity of PfKelch13 with human Keap1 and its subcellular localization suggest PfKelch13 promotes PfPI3K ubiquitination as part of an antioxidant and cytoprotective response induced during conditions of vigorous digestion of Hb.

PtdIns3P in *P. falciparum* also localizes to the apicoplast (Figure 4B), where it might act as a targeting signal for membrane transport (Tawk et al., 2010). In *T. gondii*, PtdIns3P is not associated with Rab5-positive vesicles or endosome-like structures. Rather, the major PtdIns3P-containing subcellular compartments in *T. gondii* are the apicoplast outer membrane and adjacent vesicles (Figure 4C) (Tawk et al., 2011). The distinctions in PtdIns3P localization in these two parasites likely reflect differences in their endocytic trafficking pathways. Unlike *Plasmodium*, endocytosis in *T. gondii* occurs during intracellular and extracellular parasite stages, and it is proposed to intersect with exocytic trafficking pathway(s) (Gras et al., 2019; McGovern et al., 2018; Duo et al., 2014). This model posits that endocytosis occurs at membrane invaginations distinct from the micropore (a structure similar to the cytostome) and that the endocytosed material is sorted in an endocytic-like compartment (ELC). The ELC is marked by Rab5 and Rab7 (Tomavo et al., 2013), and with plant-like vacuole proteins (Parussini et al., 2010). The ELC also represents the maturation center for microneme (Harper et al., 2006) and rhoptry secretory proteins (McGovern et al., 2018). Further analyses of how PtdIns3P pools are specified in the *T. gondii* endocytic system are required for understanding how endocytic and exocytic pathways have been repurposed in these parasites and how they intersect.

The function of PtdIns3P in the *T. gondii* apicoplast outer membrane and surrounding vesicles was analyzed by studying the effects of prolonged expression of the PtdIns3P biosensor 2xFYVE domain of the mammalian Hrs protein. Sustained PtdIns3P pool sequestration results in loss of the apicoplast, and this event ultimately leads to parasite death. These data suggest that PtdIns3P promotes apicoplast maintenance by potentiating the fusion of vesicles carrying nuclear-encoded apicoplast protein cargo with the outermost membrane of the apicoplast (FtsH1 and APT1) (Tawk et al., 2011). PtdIns3P is also involved in apicoplast inheritance/biogenesis in daughter cells through interaction with Tg/PfAtg18 to regulate Tg/PfAtg8 conjugation (Bansal et al., 2017). In *T. gondii*, this is a critical step that might precede TgAtg8 translocation to the apicoplast. This translocation step occurs upon the activation of the TgAtg12–TgAtg5–TgAtg16L complex and involves vesicular fusion in a Qbc soluble N-ethylmaleimide sensitive factor attachment protein receptor (Qbc SNARE)-dependent manner (Fu et al., 2022). Furthermore, ablation of TgPI3K, TgPIKfyve, and TgArPIKfyve activities recapitulate apicoplast loss and associated cell death, suggesting that both lipid kinases act in the same biosynthetic pathway where PtdIns3P serves as a precursor for synthesis of PtdIns(3,5)P_2_ (Daher et al., 2015). These findings reveal novel roles for PtdIns3P and PtdIns(3,5)P_2_ in intracellular vesicular trafficking with the apicoplast as the target organelle.

## The case for higher-order PtdIns3P lipids in Apicomplexa

**FIGURE 5:**
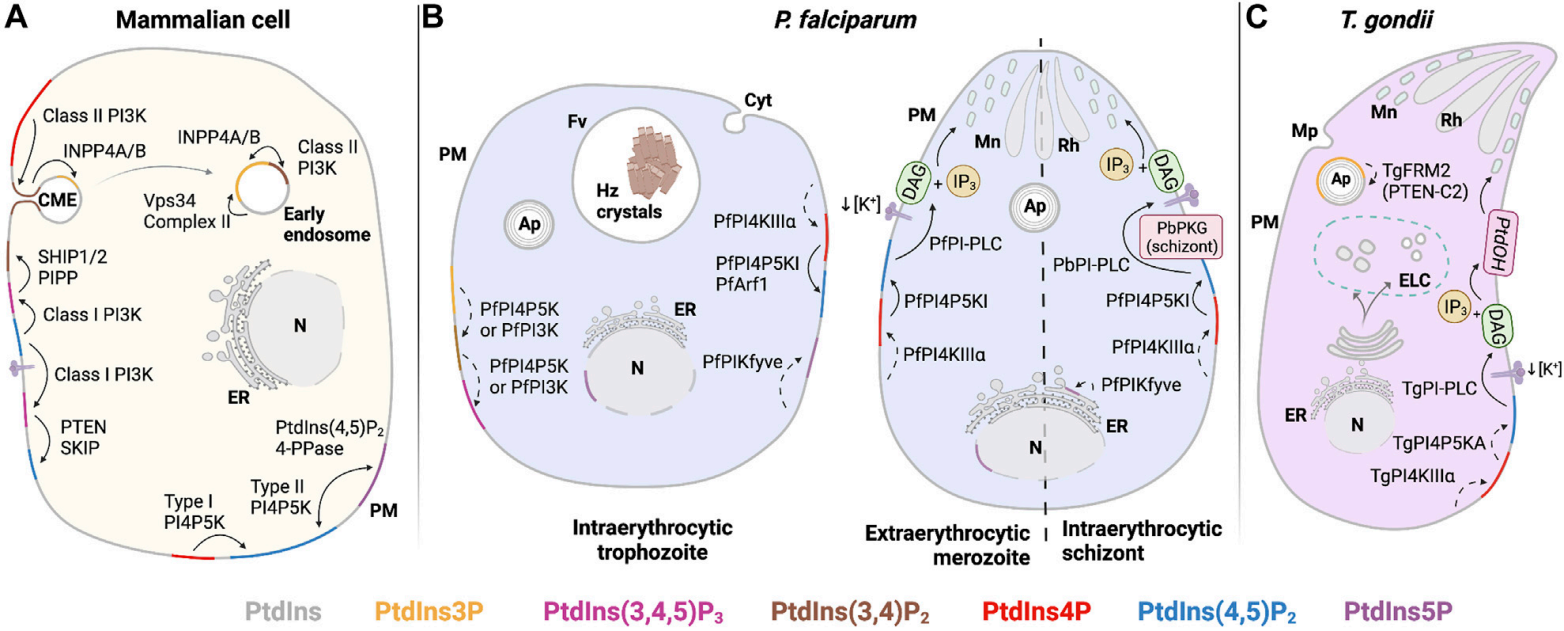
PIP regulation in the plasma membrane (PM). **(A)** Mammalian: PtdIns(4,5)P_2_ is mainly produced at the PM by phosphorylation of PtdIns4P by Type I PI4P5K. Stimulation of tyrosine kinase- and/or G-protein-coupled receptors lead to class I PI3K activation and conversion of PtdIns(4,5)P_2_ to PtdIns(3,4,5)P_3_. Dephosphorylation of PtdIns(3,4,5)P_3_ by PTEN forms PtdIns(4,5)P_2_ and turns off the signaling relay, while dephosphorylation of PtdIns(3,4,5)P_3_ by SHIP1/2 generates PtdIns(3,4)P_2_ to promote clathrin-mediated endocytosis. PtdIns5P is found at the PM after dephosphorylation PtdIns(4,5)P_2_ by a PIP_2_-4-phosphatase. **(B)** *P. falciparum*: PtdIns(4,5)P_2_ in PM is generated from PtdIns4P by type I PI4P5K after recruitment/activation by PfArf1. PtdIns(4,5)P_2_ is hydrolyzed by PfPI-PLC to produce secondary messengers, DAG and IP_3_. In *P. berghei*, positive regulation of PbPI-PLC occurs after activation of PbPKG and/or exposure to specific environmental queues (i.e., decrease of extracellular [K^+^]) to trigger microneme secretion. PtdIns(3,4,5)P_3_ may be generated by consecutive phosphorylation of PtdIns(3,4)P_2_ at OH-4 and OH-5 positions by PfPI4P5K. PtdIns5P, found at PM and nucleus in trophozoites, as well as at the ER in schizonts, may be formed by PfPIKfyve-dependent PtdIns phosphorylation. **(C)** *T. gondii*: TgPI-PLC hydrolyzes PtdIns(4,5)P_2_ to generate DAG and IP_3_. DAG is phosphorylated to PtdOH and triggers microneme exocytosis. *Solid line:* proposed reaction in previous publications and *dashed line:* proposed reaction in this review. Abbreviations in Figures 4–6: PM: plasma membrane; ER: endoplasmic reticulum; N: nucleus; Cyt: cytostome; Mp: micropore; Mc: microneme; Rh: Rhotry; Fv: food vacuole; Hz crystals: hemozoin crystals; Ap: apicoplast; DG: dense granule; ELC: endocytic-like compartment.

*In vivo* functions for PtdIns(3,4,5)P_3_ have primarily been studied in mammalian cells, where it exists at very low levels in quiescent cells. This PIP functions to recruit proteins with PtdIns(3,4,5)P_3_ - selective PH domains to the plasma membrane. Such effectors include the Akt protein kinase (protein kinase B) and phosphoinositide-dependent kinase-1 (PDK1). This process promotes cell proliferation and survival and regulates cytoskeleton dynamics, motility, membrane trafficking, and apoptosis (Cantley, 2002; Toyoshima et al., 2007; Tengholm and Idevall-Hagren, 2009). PtdIns(3,4,5)P_3_ synthesis is triggered by stimulation of tyrosine kinase- and/or G-protein-coupled receptors that activate class I PI3K (Figure 5A). The activated kinase phosphorylates PtdIns(4,5)P_2_ in the plasma membrane and elevates PtdIns(3,4,5)P_3_ levels as much as 100-fold (Tengholm and Idevall-Hagren, 2009; Vanhaesebroeck et al., 2010). An alternative pathway to generate PtdIns(3,4,5)P_3_ has been described in human platelet cells, mouse fibroblasts, and plant cells (Cunningham et al., 1990; Brearley and Hanke, 1993). This pathway involves PIP5KII-dependent phosphorylation of PtdIns3P to form PtdIns(3,4)P_2_ with subsequent PIP5KI-dependent phosphorylation of PtdIns(3,4)P_2_ to PtdIns(3,4,5)P_3_ (Zhang et al., 1997).

PtdIns(3,4,5)P_3_ has not been detected in Apicomplexans (Ebrahimzadeh et al., 2018). However, studies in *P. falciparum*-infected erythrocytes show an incremental increase of PtdIns(3,4,5)P_3_ in erythrocytic-infected states (Figure 2A) (Tawk et al., 2010). The sole PfPI3K forms PtdIns(3,4,5)P_3_ from its precursor PtdIns(4,5)P_2_ *in vitro* (Vaid et al., 2010). Primary structure comparisons show PfPI3K shares some structural features with the catalytic and helical domains of the human class I PI3K PIK3CG. These comparisons suggest that PfPI3K might produce PtdIns(3,4)P_2_ using a PtdIns4P substrate and PtdIns(3,4,5)P_3_ from a PtdIns(4,5)P_2_ precursor *in vitro,* in addition to producing PtdIns3P (Vaid et al., 2010). However, when parasites are treated with wortmannin, a PI3K inhibitor, only levels of PtdIns3P in infected erythrocytes are diminished, a result that suggests that PfPI3K produces exclusively PtdIns3P *in vivo* (Tawk et al., 2010). Thus, Tawk and colleagues (2010) propose that *P. falciparum* may harbor a similar PtdIns(3,4,5)P_3_ production pathway to the one described in the fission yeast *Schizosaccharomyces pombe*, where PtdIns(3,4,5)P_3_ synthesis is mediated by consecutive phosphorylation at the D4 and D5 positions of PtdIns3P by a PI4P5K (Figure 5B) (Mitra et al., 2004). As mentioned previously, this route corresponds to the so-called alternate synthesis pathway in multicellular organisms. This pathway might not only support PtdIns(3,4,5)*P*_3_ production in *P. falciparum*-infected erythrocytes, but it also suggests the form of an ancestral pathway for PtdIns(3,4,5)P_3_ synthesis that precedes functional diversification of the PI3K family.

PtdIns(3,4,5)*P*_3_ signals are terminated via dephosphorylation of the D3-position to form PtdIns(4,5)P_2_. Among these negative regulators of the PI3K axis in mammals are the enzymes phosphatase and tensin homolog deleted on chromosome 10 (PTEN) and skeletal muscle and kidney-enriched inositol phosphatase (SKIP) (Figure 5A) (Ono et al., 2001; Ijuin and Takenawa, 2003). By contrast, 5-phosphatases that generate PtdIns(3,4)P_2_, such as the Src-homology 2 containing inositol phosphatases 1 and 2 (SHIP1 and SHIP2), and the poorly characterized proline-rich inositol polyphosphate 5-phosphatase (PIPP), do not suppress PtdIns(3,4,5)P_3_ signaling (Astle et al., 2006; Pedicone et al., 2021; Tengholm and Idevall-Hagren, 2009).

The PTEN-C2 domain has only been described as an accessory domain in the formin 2 proteins of *P. falciparum*, *P. cynomolgi*, *P. knowlesi*, *P. vivax*, and *T. gondii* (Stortz et al., 2019; Pathak et al., 2019). Formin is a multidomain protein that is an important regulator of actin polymerization in eukaryotes. The PTEN-C2 domain-containing formin 2 (FRM2) localizes adjacent to the apicoplast in both *P. falciparum* and *T. gondii* (Figures 4B,C). Conditional disruption of the gene encoding PfFRM2 and TgFRM2 results in structurally aberrant apicoplasts, loss of cell viability in *P. falciparum* (the only case studied thus far), deranged F-actin network dynamics, and impaired cytokinesis (Stortz et al., 2019). These phenotypes recapitulate those that accompany diminutions in PtdIns3P and PtdIns(3,5)P_2_ synthesis in both Apicomplexans (Tawk et al., 2010; Daher et al., 2015). These collective observations raise interesting questions regarding whether PfFRM2 and TgFRM2 function is linked to apicoplast biogenesis/maintenance by PTEN-C2 domain-dependent regulation of PtdIns(3,4,5)P_3_, although the presence of PtdIns(3,4,5)P_3_ in this compartment has not been confirmed. Alternatively, the PTEN-C2 domain of PfFRM2 and TgFRM2 might sense PtdIns3P and PtdIns(3,5)P_2_. PlasmoDB(v56) and ToxoDB(v56) databases identify common homologs for SHIP1, SHIP2, SKIP, and PIPP phosphatases: PF3D7_0705500 and TGME49_238400 (Supplementary Table S1). Both genes are essential for parasite viability and are annotated as putative inositol polyphosphate-related phosphatases. These represent candidate 5-phosphatases that regulate PtdIns(3,4,5)P_3_ signaling in both organisms.

As in the case of PtdIns(3,4,5)P_3_ in *P. falciparum*, PtdIns(3,4)P_2_ has been detected exclusively in infected erythrocytes, and its production via phosphorylation of PtdIns4P by the sole PfPI3K isoform has only been demonstrated *in vitro* (Tawk et al., 2010; Vaid et al., 2010). In *T. gondii*, the PtdIns(3,4)P_2_-selective PH domain of human TAPP1 localizes to the plasma membrane in intracellular tachyzoites (Arabiotorre et al., 2023). This result indicates the existence of a persistent PtdIns(3,4)P_2_ pool in this compartment. Apicomplexan genome database annotations do not identify ortholog candidate class II and class III PI3-kinases, which produce PtdIns(3,4)P_2_ and PtdIns(3,4,5)P_3_ in higher organisms (Brown and Auger, 2011). Thus, as suggested previously, PtdIns(3,4)P_2_ might be produced by phosphorylation at D4 of PtdIns3P by a PI4P5K (Mitra et al., 2004; Tawk et al., 2010). In mammals, downregulation of PtdIns(3,4)P_2_ signaling occurs primarily via the action of members of the 3- and 4-phosphatase protein families also involved in PtdIns(3,4,5)P_3_ degradation. Candidate orthologs for specific PtdIns(3,4)P_2_ phosphatases such as the 4-phosphatases INPP4A and INPP4B are not present in Apicomplexa. Hence, there is little evidence for the existence of regulatory proteins involved in producing or consuming PtdIns(3,4)P_2_ in the Apicomplexa.

## Apicomplexan PtdIns-4-OH kinases

PtdIns4P regulates membrane trafficking through the TGN/endosomal system, lipid homeostasis, autophagy, cytokinesis, and actin dynamics in mammals and yeast (Rivas et al., 1999; Hama et al., 1999; Walch-Solimena and Novick, 1999 Jović et al., 2012; D’Angelo et al., 2007; Wang et al., 2015; Sechi et al., 2014). In this section, we discuss synthesis of PtdIns4P, mechanisms of its regulation, and its role in signaling processes in Apicomplexa. PtdIns4P is produced by PtdIns 4-OH kinases (PI4Ks) and is degraded by PtdIns4P phosphatases. PI4Ks are classified in two structural classes: 1) type II kinases represented by PI4KIIα and PI4KIIβ in mammals and Lsb6 in yeast and 2) type III kinases represented by PI4KIIIα and PI4KIIIβ in mammals and their respective yeast orthologs Stt4 and Pik1 (Han et al., 2002; Strahl and Thorner, 2007; Clayton et al., 2013). Candidate PI4Ks are listed in both *P. falciparum* and *T. gondii* genome databases (Wengelnik et al., 2018). There are three PI4K candidates in *P. falciparum*: PfPI4KII, PfPI4KIIIα, and PfPI4KIIIβ. *T. gondii* encodes three orthologs of the *P. falciparum* proteins (TgPI4KII, TgPI4KIIIα, and TgPI4KIIIβ) and four putative “PI3K/PI4K-like” proteins. It is likely that these “PI3K/PI4K-like” proteins are not true PtdIns kinases. In all four cases, the human and yeast proteins with the highest similarity represent protein kinases. These include Tor2/mTOR (homologous to the product of TGME49_316430). Thus, we will not further discuss those kinases in this review.

Type II PI4Ks are dispensable for viability in yeast and Metazoa, yet these enzymes carry out specific cellular functions. In mammals, PI4KIIα and PI4KIIβ are associated with endosomal compartments (Balla et al., 2002; Wei et al., 2002; Jović et al., 2012). In yeast, Lsb6 regulates endosome motility in a manner that is apparently independent of its catalytic activity (Chang et al., 2005). Phylogenetic analyses of PIP lipid kinases indicate that *Plasmodium* type II PI4K is more closely related to the plant type II PI4Ks than to metazoan or yeast isoforms. This relationship likely reflects lateral gene transfer from a red algal endosymbiont (Brown and Auger, 2011; McFadden, 2011). Genes annotated to encode type II PI4K-like proteins are listed in *Plasmodium* and *T. gondii* genomes. Although these proteins appear to confer fitness to these organisms, their activities and functions remain uncharacterized. For these reasons, we focus discussion on Type III PI4Ks (Sidik et al., 2016; Bushell et al., 2017).

### PtdIns4P synthesis in the Apicomplexan secretory pathway

**FIGURE 6:**
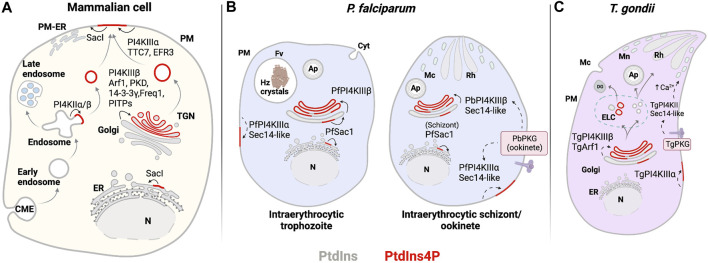
PIPs involved in exocytosis. **(A)** Mammalian: PtdIns4P is produced in the TGN and the PM by PI4KIIIβ and PI4KIIIα. PI4KIIIα is recruited to the PM and activated by TTC7 and EFR3. At the TGN, PI4KIIIβ is recruited by Freq1 and activated by PKD and 14-3-3γ, while PITPs improve PI4KIIIβ inefficient activity. Recycling of molecules from endosomes through exocytosis follows synthesis of PtdIns4P by PI4KIIα/β at the endosomes. Degradation of PtdIns4P occurs through activity of the phosphatase Sac1 in the PM and at the Golgi and ER membranes. **(B)** *Plasmodium*: PtdIns4P is produced at the Golgi complex by PfPI4KIIIβ, where it regulates membrane trafficking events during trophozoite and schizont stages. PbPKG positively regulated PfPI4KIIIβ in ookinetes. PfSacI negatively regulated PtdIns4P levels occurring at the ER. **(C)** *T. gondii*: PtdIns4P is expected to be found at the Golgi complex and potentially generated by TgPI4KIIIβ and TgArf1. *Solid line:* proposed reaction in previous publications and *dashed line:* proposed reaction in this review. Abbreviations in Figures 4–6: PM: plasma membrane; ER: endoplasmic reticulum; N: nucleus; Cyt: cytostome; Mp: micropore; Mc: microneme; Rh: Rhotry; Fv: food vacuole; Hz crystals: hemozoin crystals; Ap: apicoplast; DG: dense granule; ELC: endocytic-like compartment.

PfPI4KIIIβ (PF3D7_0509800) is the sole Apicomplexan enzyme directly demonstrated to have PI4K activity (McNamara et al., 2013; Stenberg and Roepe, 2020). Sequence alignment and secondary structure predictions suggest that PfPI4KIIIβ domain organization is similar to that of human PI4KIIIβ (UniProt Q9UBF8), including helical N- and C-lobes and a conserved catalytic domain. PfPI4KIIIβ is twice as large as its human ortholog due to expansion of sequences of unknown significance that separate the conserved domains (Figure 3). The lipid kinase activity of the PfPI4KIIIβ catalytic domain and the C-terminal portion of the N-lobe (Km_ATP_ = 72.5 μM) is comparable with the lipid kinase activities of human PI4KIIIα and PI4KIIIβ (70 μMand 90 μM, respectively) (Sternberg and Roepe, 2020). This kinase likely executes essential functions at all stages of the parasitic life cycle in vertebrate hosts (McNamara et al., 2013; Bushell et al., 2017). The *P. berghei* PI4KIIIβ (PBANKA_1109400) is phosphorylated and potentially regulated by the protein kinase PbPKG (Figure 6B) and has a role in parasite gliding motility. However, it is not known if the lipid kinase activity is required in this context. A mutant strain lacking consensus PKG-dependent phosphorylation sites in PbPI4KIIIβ (*pi4k* ^*S534A*^ and *pi4k* ^*S534A/S538A*^) exhibits a significant decrease in gliding speed compared with control lines (Brochet et al., 2014).

PfPI4PKIIIβ is distributed throughout the cytosol in trophozoites and is enriched at the apical ends of developing daughter merozoites (McNamara et al., 2013). PtdIns4P pools have been detected at the Golgi complex by colocalization with the marker ER Retention Defective protein 2 (ERD2) and at the plasma membrane throughout the erythrocytic cycle of *P. falciparum* (Figures 6B, 2A) (McNamara et al., 2013; Ebrahimzadeh et al., 2018). Synthesis of Golgi PtdIns4P pools is sensitive to imidazopyrazine, a PfPI4KIIIβ inhibitor, suggesting that this pool is generated by PfPI4KIIIβ. The specificity of imidazopyrazine was determined by comparing its effects with those of quinoxaline, a known inhibitor of human PI4KIIIβ. Both inhibitors induce essentially the same schizont-stage arrest. Moreover, imidazopyrazine- and quinoxaline-resistant parasites carry missense substitutions either in the PfPI4KIIIβ coding gene or in genes encoding proteins whose activity is regulated by the kinase. These resistant lines show comparable cross-resistance against both inhibitors. Taken together, these data make a convincing case that PfPI4KIIIβ is the direct cellular target of imidazopyrazine (McNamara et al., 2013).

**FIGURE 7:**
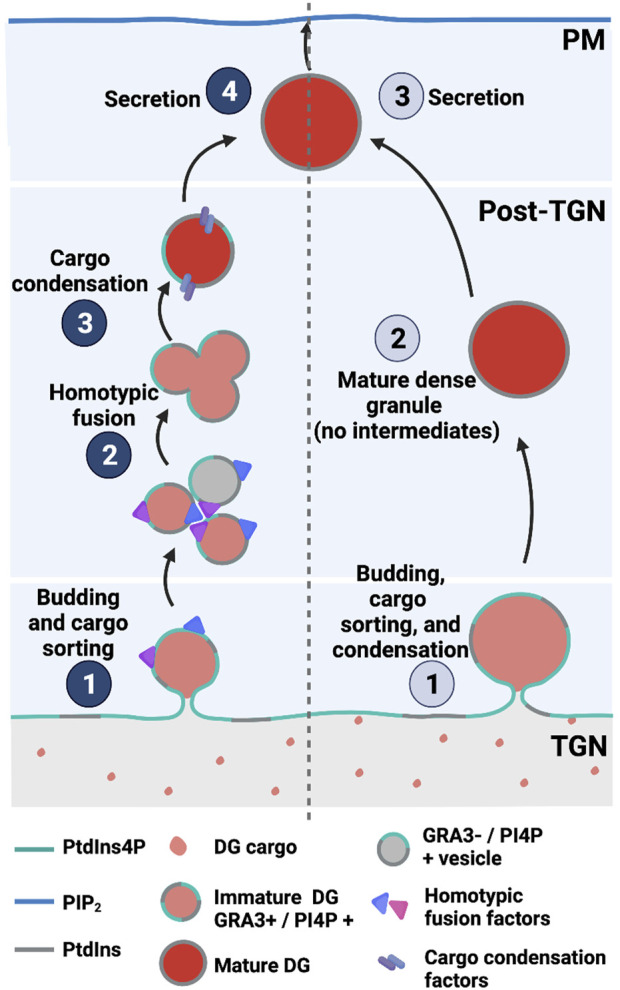
PtdIns4P-dependent dense granule multistep biogenesis/maturation model (left) compared with direct budding models (right) in *T. gondii*.

Trafficking of post-Golgi vesicles produced in a PI4KIIIβ-dependent manner target the plasma membrane and are involved in promoting cell division and cytokinesis in yeast and higher eukaryotes (Audhya et al., 2000; Schorr et al., 2001; Sechi et al., 2014). A recent study reported PtdIns4P pools in the *T. gondii* Golgi/TGN system and in post-TGN compartments. Moreover, perturbation of PtdIns4P signaling by expression of high-affinity PtdIns4P binding domains evoked defects in dense granule biogenesis (maturation?) and exocytosis (Arabiotorre et al., 2023). Those results are not easily in agreement with those of current models that envision a process where mature secretion-competent dense granules bud directly from the TGN (Griffith et al., 2022). Rather, the data indicate a role for PtdIns4P signaling in a multi-step dense granule maturation pathway that resembles those characterized for regulated secretory pathways in professional secretory cells (Figure 7).

How are PtdIns4P signals read? TgRab11A is an ortholog of the Ras-like GTPase Rab11—a known PtdIns4P effector in higher eukaryotes–and this protein plays a key role in dense granule exocytosis and protein trafficking to the plasma membrane (Venugopal et al., 2020a). Additional evidence for Rab11 in promoting downstream PtdIns4P signaling comes from studies in *P. falciparum*. In a manner similar to the case of cytokinesis in *Drosophila* spermatocytes, where deposition of a new membrane at the midzone requires PI4KIIIβ-dependent Rab11 localization at Golgi and midzone membranes (Polevoy, et al., 2009), and PfPI4KIIIβ-PfRab11A-regulated membrane trafficking facilitates cytokinesis during the intraerythrocytic stage (McNamara et al., 2013). The association of PfRab11A with PfPI4KIIIβ activity, and the coordination of Rab11A and its effectors by PI4KIIIβ on PtdIns4P-enriched membranes in *Drosophila* and humans (Polevoy, et al., 2009; Burke et al., 2014), suggests Apicomplexan PI4KIIIβ-mediated PtdIns4P production effects proper membrane recruitment of Rab11A for purposes of membrane trafficking control in *P. falciparum* and *T. gondii*.

In yeast and mammals, recruitment of Pik1 and PI4KIIIβ to the Golgi complex is facilitated by the small GTPase Arf1, the plasma membrane protein NCS-1 (Frq1 in yeast) and GGA2 (Figure 6A) (Zhao et al., 2001; Daboussi et al., 2017; Highland and Fromme, 2021). Among these regulatory components, an Arf1 homolog has been identified in Apicomplexans and is associated with trafficking processes, although a role for the Arf1 homolog in PI4KIIIβ recruitment to the Apicomplexan Golgi system has not yet been described. *T. gondii* Arf1 (TgArf1) localizes to Golgi membranes. In a permeabilized cell secretion assay, TgArf1 stimulated the secretion of preformed dense granules (Liendo et al., 2001). TgArf1 may promote PtdIns4P synthesis at the Golgi and subsequently potentiate membrane trafficking from this compartment as is the case for yeast and mammalian Arf1 orthologs (Figure 6C). *P. falciparum* Arf1 (PfArf1), however, has not yet been associated with PtdIns4P synthesis. PfArf1 localizes to the ER and is required for *Plasmodium* export element (PEXEL)-dependent and -independent protein export pathways from this compartment (Taku et al., 2021).

In mammalian cells, activation of PI4KIIIβ at the Golgi/TGN occurs via phosphorylation of Ser294 by protein kinase D (PKD) (Hausser et al., 2005). This modification is maintained by the interaction of PI4KIIIβ with the 14-3-3γ adapter protein and sustains the PtdIns4P production that supports membrane fission events (Hausser et al., 2006). In *P. falciparum* and *T. gondii*, the closest ortholog to PKD1/2 is CDPK7. In *T. gondii,* CDPK7 activity is essential for correct positioning and partitioning of the centrosomes during parasite division (Morlon-Guyot et al., 2014). Moreover, genes encoding potential 14–3-3 orthologs have been identified in *P. falciparum, P. knowlesi,* and *T. gondii*. However, their precise functions remain to be elucidated (Al-Khedery et al., 1999; Koyama et al., 2001; del Mar Siles-Lucas and Gottstein, 2003). Quantitative phosphoproteomic profiling in *T. gondii* parasites lacking TgCDPK7 shows that TgRab11A is a primary substrate for modification by TgCDPK7 and that TgCDPK7-dependent phosphorylation of TgRab11A is critical for protein trafficking, TgRab11A localization, and parasite division (Bansal et al., 2021). The association of Apicomplexan Rab11A with PI4KIIIβ regulators supports the likelihood that conserved mechanisms for PtdIns4P synthesis exist in the Golgi/TGN system of Apicomplexans.

### PITP-dependent regulation of PtdIns4P signaling in the secretory pathway

Studies in yeast reveal that PtdIns4P kinases are biologically insufficient enzymes, i.e., the levels of PtdIns4P these produce fall below the threshold required to sustain PtdIns4P signaling and maintain cell viability. This functional barrier is overcome by the activity of phosphatidylinositol transfer proteins (PITPs) which are more accurately referred to as PtdIns exchange proteins. Sec14, the major yeast PITP, exchanges PtdCho for PtdIns on membrane surfaces. It is this heterotypic exchange cycle that stimulates PI4K to phosphorylate its substrate, likely because the PtdIns head group becomes exposed from the membrane bilayer and available for kinase action (Schaaf et al., 2008; Bankaitis et al., 2010; Sugiura et al., 2019). In the yeast TGN/endosomal system, the affected kinase is Pik1 (PI4KIIIβ in mammals). Upon stimulation by the Sec14 lipid exchange cycle, Pik1 produces sufficient PtdIns4P to potentiate membrane trafficking (Hama et al., 1999; Rivas et al., 1999; Schaaf et al., 2008; Bankaitis et al., 2010).

PITPs are evolutionarily conserved proteins that fall into two structurally distinct families: the Sec14-like PITPs, of which Sec14 is the founding member, and StAR-related lipid transfer (StART) PITPs (Sha et al., 1998; Yoder et al., 2001; Schaaf et al., 2008). These PITP families are structurally unrelated, but both include members that regulate PtdIns 4-OH kinase activities through the heterotypic lipid exchange reaction between PtdIns and a secondary ligand (Schaaf et al., 2008; Bankaitis et al., 2010; Xie et al., 2018). The identity of the secondary ligand serves as signaling input into the PITP exchange lipid cycle. For example, PtdCho is the secondary ligand of Sec14, and one of PtdCho biosynthetic pathways is represented by the CDP–choline pathway, which consumes DAG. Moreover, adequate levels of DAG in TGN/endosomal membranes are necessary to initiate vesicle budding and exocytosis. When sufficient DAG levels are available for vesicle biogenesis at the TGN/endosomes, and to be used as a substrate for PtdCho production, Sec14 senses PtdCho production levels as metabolic information to drive PtdIns4P synthesis.

**FIGURE 8:**
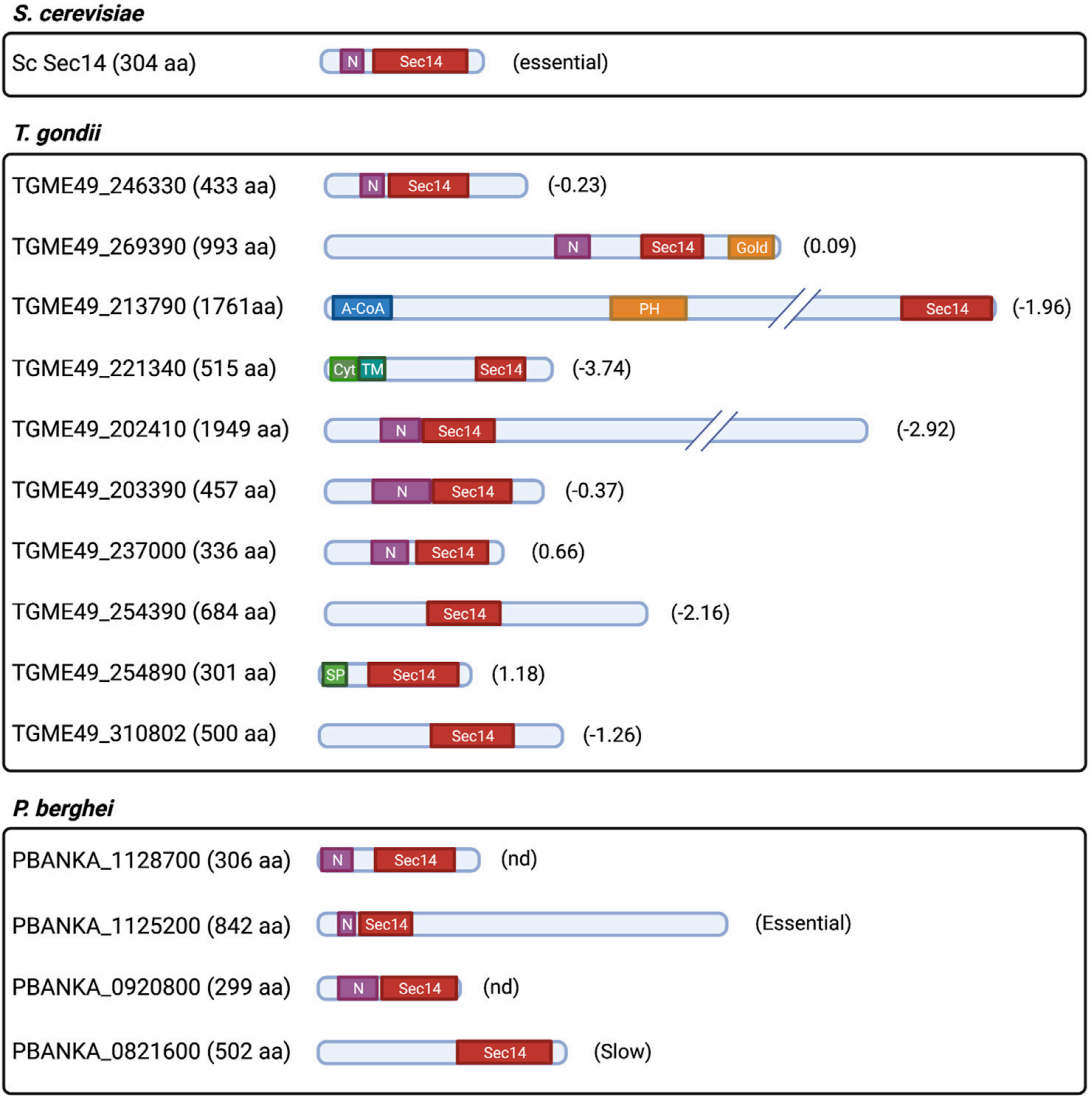
Prediction of protein architecture of Sec14-like protein candidates in *T. gondii* and *P. falciparum.* ScSec14p is depicted as the founding member of the Sec14-like protein family. Domain architecture of *T. gondii* and *P. falciparum* protein candidates that were selected upon prediction of the CRAL-TRIO domain through the Interpro domain finding tool in PlasmoDB (v56) and ToxoDB (v56). N: N-term CRAL-TRIO; Gold: Golgi dynamics domain; A-CoA: Acyl-CoA binding domain; PH: Pleckstrin homology domain; Cyt: Cytoplasmic domain; TM: Transmembrane domain; SP: Signal peptide; and (#): gene phenotypic score obtained from Sidik et al., 2016 for *T. gondii*; and relative growth rate score obtained from Bushell et al., 2016 for *P. falciparum* gene candidates (Essential: essential for growth; slow: significant slow growth rate; and nd: not determined).

Apicomplexans encode both Sec14- and StART-like proteins (Supplementary Table S1). *Plasmodium* and *T. gondii* encode four and 10 proteins containing the Sec14 domain (also referred as the CRAL-TRIO domain with a N-CRAL-TRIO region), respectively. The protein architecture and sizes of *T. gondii* Sec14-like proteins are more diverse than what is observed in yeast and in *P. falciparum* (Figure 8). At least three of these candidates are multidomain proteins of some 2,000 amino acids. Yeast encodes six single-domain Sec14-like proteins of similar size (Wu et al., 2000; Li et al., 2002; Routt et al., 2005; Ren et al., 2011; Kotomura et al., 2018), and only Sec14p is essential for cell viability (Bankaitis et al., 1989). The biological requirement of Sec14-like genes in *T. gondii* is also distinct as five of 10 of these genes are required for parasite survival (Sidik et al., 2016). These observations suggest that Sec14-like proteins are involved in diverse functions in *T. gondii* parasites.

Although Apicomplexan Sec14-like proteins are yet to be functionally characterized, there are indirect indications that some of these likely mediate PtdIns4P metabolism. For instance, mutations in a putative PfPI4KIIIα (PF3D7_0419900) and PfSec14-like (PF3D7_0626400) proteins were the primary contributors to positive fitness in *P. falciparum* kelch13 loss-of-function mutants (Cerqueira et al., 2017). As described previously, PtdIns3P levels are regulated by PfKelch13 (Mbengue et al., 2015). These results suggest that PfPI4KIIIα (PF3D7_0419900) and PfSec14-like (PF3D7_0626400) candidates normalize PtdIns3P metabolism/signaling in parasites lacking functional PfKelch13. An independent phosphoproteomic study in *P. berghei* ookinetes linked PbPI4KIIIα (PBANKA_0722000), PbSec14-like protein (PBANKA_1125200), and PbPI4KIIIβ (PBANKA_1109400) to a phosphoinositide (PtdIns4P/PtdIns(4,5)P_2_) signaling pathway that responds to an endogenous cyclicGMP-dependent protein kinase (PKG) involved in intracellular Ca^2+^ mobilization (Brochet et al., 2014). In addition, two independent *T. gondii* phosphoproteomic analyses reported that a Sec14-like protein (TGME49_254390) (Nofal et al., 2022) and a putative TgPI4KII (TGME49_276170) (Herneisen et al., 2022) are phosphorylated targets of Ca^2+^-dependent protein kinases (TgCDPK) and TgPKG, respectively. Collectively, these data suggest the intriguing possibility that Sec14-like protein-dependent PtdIns4P production is part of a cGMP/Ca^2+^ pathway that modulates microneme secretion and parasite egress in Apicomplexans.

A StART-like PITP has been described in *P. falciparum*. This essential PfStART-like protein (PF3D7_0104200) is present in the PV (in trophozoite stages) and in small compartments surrounding the edge of the parasite (in mature schizonts). Lipid transfer assays show that PF3D7_0104200 binds and transfers PtdIns and PtdCho between membranes *in vitro* at levels comparable to those measured for rat PITPα. It was suggested this protein is involved in acquisition of phospholipids from the host cell (van Ooij et al., 2013; Hill et al., 2016). However, the possibility that it might stimulate PIP synthesis more directly via a heterotypic exchange cycle has not been addressed. *T. gondii* also encodes a single-candidate StART-like PITP (TgME49_289570), which is predicted to represent a multi-domain protein that contains an N-terminal PITP domain, a PH domain, and a C-terminal OSBP domain. This gene has not been studied directly, but it serves as a likely essential gene in a CRISPR-based phenotypic screen (Sidik et al., 2016).

### Regulators of PtdIns4P production at the plasma membrane

PtdIns4P can be delivered to the plasma membrane by vesicular carriers from the Golgi complex and from recycling endosomes (Dickson et al., 2014). PtdIns4P is also directly produced in the plasma membrane by PI4KIIIα isoforms, e.g., Stt4 in yeast and the mammalian ortholog PI4KIIIα (Figure 6A). Recruitment and activation of these kinases in the plasma membrane occurs via interaction with soluble TTC7 and transmembrane EFR3 proteins that represent homologs of the yeast Ypp1 and Efr3 proteins, respectively (Audhya et al., 2000; Nakatsu et al., 2012; Batrouni and Baskin, 2021). Both *Plasmodium* and *T. gondii* encode single-candidate PI4KIIIα-like proteins (PF3D7_0419900 and TGGT1_228690) (Wengelnik et al., 2018). However, no obvious candidate orthologs are annotated for any of the known PI4KIIIα/Stt4 accessory proteins. As described previously, PtdIns4P was detected at the plasma membrane throughout the erythrocytic cycle of *P. falciparum* (Ebrahimzadeh et al., 2018). Hence, recruitment of PI4KIIIα to the Apicomplexan plasma membrane, and production of PtdIns4P at that site, might occur via a novel mechanism.

### Downregulation of PtdIns4P signaling in Apicomplexa

Downregulation of PtdIns4P signaling is primarily executed by the highly conserved PtdIns4P-phosphatase Sac1. This enzyme shows substrate promiscuity *in vitro* but primarily dephosphorylates the 4 position of the inositol ring to produce PtdIns at the expense of PtdIns4P *in vivo* (Guo et al., 1999; Rivas et al., 1999). Sac1 is enriched at the Golgi and ER membranes of budding yeast and metazoan organisms and is proposed to establish a PtdIns4P gradient across the Golgi complex with the TGN harboring the highest level of PtdIns4P (Piao and Mayinger, 2012; Wood et al., 2012; Venditti et al., 2016; Del Bel and Bril, 2018). In addition, Sac1 is proposed to degrade PtdIns4P derived from the plasma membrane at ER–plasma membrane contact sites (Figure 6A) (Chung et al., 2015). A *P. falciparum* Sac1 (PfSac1: PF3D7_1354200) contains the conserved CX5R(T/S) motif essential for catalytic activity (Liu and Bankaitis, 2010; Thériault and Richard, 2017). This PfSac1 is expressed in all asexual blood stages and is likely required for survival during the erythrocytic cycle. PfSac1 also localizes to the ER and Golgi membranes (Figure 6B), whereas localization to both organelles becomes less apparent during early schizont stages. Yeast and mammalian Sac1 shuttle between the ER and Golgi in a growth phase-dependent manner. Although Sac1 concentrates at the ER in dividing cells, it translocates to the Golgi complex in resting cells or under starvation conditions (Whitters et al., 1993; Blagoveshchenskaya et al., 2008). On that basis, it is suggested that PfSac1 disengages from the Golgi system in schizonts and distributes preferentially to transitional ER (Thériault and Richard, 2017). Whether PfSac1 localization is subject to regulation similar to that observed in yeast, perhaps to control the expansion of PtdIns4P-rich domains in the Golgi complex during the progression of parasite erythrocytic life cycle, remains to be determined.

In yeast, Sec14-dependent PtdIns4P signaling is also downregulated by one of the seven members of the oxysterol-binding protein (OSBP) superfamily, Kes1/Osh4 (Fang et al., 1996). Kes1/Osh4 is proposed to act as a sterol-regulated ‘brake’ of TGN/endosomal trafficking via its ability to clamp/sequester Pik1-generated Golgi PtdIns4P from its effectors (Mousley et al., 2012). Genomic annotations predict one OSBP/ORP homolog in *Plasmodium* and three in *T. gondii* (Supplementary Table S1). However, these proteins have not been characterized. As described in the previous section, the single-candidate StART-like PITP in *T. gondii* is a predicted multidomain protein with a C-terminal OSBP-like domain. Although neither the PITP nor the OSBP activities of these domains has been confirmed, this architecture presents a physical coupling of two domains that exhibit functional antagonism in regulating PtdIns4P signaling. This curious point suggests that the relationship between PITP activity and OSBP antagonism described in yeast might have evolved in most ancient eukaryotes. Whether that antagonism operates through regulation of PI4P signaling in *T. gondii* remains unknown.

Two OSBP/ORP proteins have been identified and characterized in *C. parvum* (CpORP1 and CpORP2). Cholesterol is not a binding ligand for either protein, whereas PtdOH and PtdIns monophosphates (PtdIns3P, PtdIns4P, and PtdIns5P) are bound with high affinity. CpORP1 localizes to the PVM, suggestive of a lipid transport role across the interface between the parasite and the host intestinal lumen (Zeng et al., 2006). Alternatively, CpORP1 may be involved in PIP sensing at the PVM.

Few factors associated with synthesis and regulation of PtdIns4P levels in *Plasmodium* have been described, and there is a complete lack of information regarding PtdIns4P metabolism in Apicomplexan organisms. As reviewed, PtdIns4P distributes to the Golgi complex where it executes important regulatory roles in vesicle trafficking and secretion, and to the plasma membrane. Contrary to protein regulators associated with PtdIns4P synthesis in the plasma membrane, almost every such regulator associated with PtdIns4P in the Golgi complex (discussed in this section) has at least one candidate homolog in *Plasmodium* and *T. gondii* (Supplementary Table S1). Hence, addressing the link between PtdIns4P metabolism in the Golgi complex and trafficking of specialized Apicomplexa secretory proteins (microneme, rhoptry, and dense granule proteins) identifies an important step in understanding ancient and conserved intracellular trafficking and cell secretion processes.

## PtdIns(4,5)P_2_ in Apicomplexa

PtdIns(4,5)P_2_ is primarily concentrated at the plasma membrane of eukaryotic cells and contributes to many activities that involve the plasma membrane. These include actin cytoskeleton assembly, cell polarization, cell migration, endocytic vesicle formation and scission, exocytic vesicle fusion to the plasma membrane, and ion channel and transporter activation. Upon receptor stimulation, PtdIns(4,5)P_2_ is metabolized to produce second messengers such as PtdIns(3,4,5)P_3_, inositol trisphosphate (IP_3_ or I(1,4,5)P_3_), DAG, phosphatidic acid (PtdOH), arachidonic acid (AA), and lysophospholipids that further propagate and amplify signal transduction (Di Paolo and De Camilli, 2006; De Craene et al., 2017; Wen et al., 2021). In mammals, the major pathway for producing PtdIns(4,5)P_2_ in the plasma membrane is by phosphorylation of PtdIns4P by a type I PI4P5K (PIP5K1-α, PIP5K1-β and PIP5K1-γ) (Figure 5A). In multicellular organisms, a second type II PI5P4K phosphorylates PtdIns5P to produce PtdIns(4,5)P_2_ in the Golgi complex. In yeast, the type I PI4P5K (Mss4p) is the only PIP kinase that generates PtdIns(4,5)P_2_, and it is essential for cell viability (Desrivières et al., 1998; Ishihara et al., 1998; Hinchliffe and Irvine, 2010; Rameh, 2010).

PtdIns(4,5)P_2_ is by far the most abundant PIP in *P. falciparum* (Tawk et al., 2010). PtdIns(4,5)P_2_ is detected at the plasma membrane throughout all stages of the cell cycle (Figure 2A) and, during the ring stages, in membranes of large vesicles that likely represent endocytic compartments (Figure 4B) (Ebrahimzadeh et al., 2018). A putative bifunctional PIP5K was identified in *P. falciparum* (PF3D7_0110600) that contains a C-terminal type I PI4P5K domain with catalytic specificity for PtdIns4P, and an N-terminal domain with potential helix-loop-helix EF-hand-like motifs found in members of the NCS family (NCS-EF domain) (Figure 3). This PfPI4P5K preferentially phosphorylates PtdIns4P and is stimulated by myristoylated Arf1 *in vitro* (Leber et al., 2009). The physical association of type I PI4P5K and NCS-EF domains is unique to Apicomplexa. This property could reflect an exclusive modulation of the PtdIns(4,5)P_2_ synthesis in response to changes in intracellular calcium concentrations within the parasite. Unlike *Plasmodium*, which only encodes a single putative PI4P5K isoform, *T. gondii* encodes two isoforms of PI4P5K termed TgPI4P5KA and TgPI4P5KB (TGME49_230490 and TGME49_245730, respectively) (Wengelnik et al., 2018). Only TgPI4P5KA is likely to be essential for parasite viability (Sidik et al., 2016), and this enzyme exhibits an EF-hand motif. This organization recommends this isoform as the true PfPI4P5K ortholog and that it is involved in crosstalk between Ca^2+^ signaling and PIP metabolism. In that regard, PtdIns(4,5)P_2_ pools have been detected at the *T. gondii* plasma membrane using a 2xPHPLCδ reporter (Arabiotorre et al., 2023).

### Regulation of PtdIns(4,5)P_2_ levels in Apicomplexa

PtdOH is a potent stimulator of type I PI4P5Ks in mammals and is generated in part by phospholipase D (PLD)-mediated degradation of PtdCho. PLD in mammals are involved in a complex inter-regulatory relationship with type I PI4P5K (Divecha et al., 2000; Jones et al., 2000; Hinchliffe and Invine, 2010). However, PfPI4P5K is not stimulated by PtdOH *in vitro* (Leber et al., 2009). PLD-like proteins have not been identified in either *T. gondii* or in *Plasmodium* genome databases, a finding consistent with the lack of regulation of PfPI4P5K by PtdOH. Perhaps, PtdOH-dependent stimulation of type I PI4P5K was acquired in multicellular organisms later in the evolutionary process.

In mammals and yeast, PtdIns(4,5)P_2_ steady-state levels are subject to negative regulation by synaptojanin-like proteins (5-phosphatases) that degrade the PtdIns(4,5)P_2_ and downregulate signaling on internal membranes. In metazoans, these enzymes regulate the synaptic vesicle cycle and, in yeast, these are linked to endocytosis and actin dynamics (Stefan et al., 2002; Verstreken et al., 2003). Proteins homologous to synaptojanins 1 and 2 are encoded in *Plasmodium* and *T. gondii* genomes (Supplementary Table S1), respectively. These proteins have not been extensively studied in Apicomplexa. However, one and two of these putative homologs in *Plasmodium* and *T. gondii*, respectively, play essential roles in infective stages (Sidik et al., 2016; Bushell et al., 2017).

### The role of PtdIns(4,5)P_2_ in parasite invasion, egress, and motility

The hydrolysis of PtdIns(4,5)P_2_ by phospholipases generates a set of secondary messengers that transduce highly specific intracellular signals (Bunney and Katan, 2011). For instance, hydrolysis of PtdIns(4,5)P_2_ by phospholipase C (PLC) produces the plasma membrane DAG and soluble IP_3_. DAG binds and activates C1 domain-containing proteins such as protein kinase C (PKC) family at the plasma membrane, while IP_3_ diffuses into the cytosol to interact with the sarco-ER Ca^2+^ release channel (SERCA) (Streb et al., 1983; Berridge and Irvine, 1984). In mammals, there are six families of PLC enzymes (PLCβ, γ, δ, ε, ζ, and η), whereas only PLCδ is present in single-cell eukaryotes (Koyanagi et al., 1998; Bunney and Katan, 2011). *P. falciparum* and *P. berghei* harbor essential genes that encode putative phosphoinositide-specific PLC (PI-PLC) proteins: PfPI-PLC and PbPI-PLC (PF10_0132 and PBANKA_121190, respectively). These proteins bear the signatures of ancestral δ-type PLCs. Both versions contain all domains found in PLCδ: i) the lipid-binding Pleckstrin homology (PH) domain, ii) the calcium-binding motif EF-hand, iii) the catalytic domain consisting of an X- and a Y-domain, and iv) the calcium/lipid-binding C2-domain. Nonetheless, *Plasmodium* PI-PLCs are twice as large as their mammalian orthologs due to large insertions of unknown function (Raabe A. et al., 2011). *T. gondii* also encodes a single essential gene for PI-PLC (TgPI-PLC; TGME49_248830) that bears a domain structure similar to those of *Plasmodium* PI-PLCs (Bullen et al., 2016).

How PI-PLC activity is regulated in Apicomplexans is not well-understood. In *P. berghei,* PtdIns(4,5)P_2_ hydrolysis occurs in the plasma membrane, and the resulting IP_3_ triggers Ca^2+^ mobilization in the cytosol during gametocyte activation. These responses are blocked by a PI-PLC inhibitor (U73122) (Raabe A. C. et al., 2011). Exposure of *P. falciparum* merozoites to the low levels of K^+^ normally found in human plasma elevate intracellular Ca^2+^ levels that, in turn, stimulate protein release from micronemes (Figure 5B). This pathway of protein exocytosis from micronemes is inhibited by U73122. In addition, the interaction of released microneme proteins with erythrocytic receptors restores basal cytosolic Ca^2+^ levels in the parasite and triggers rhoptry secretion. This sequential discharge of micronemes and rhoptry contents in merozoites is essential for erythrocyte invasion (Singh et al., 2010). TgPI-PLC localizes to punctate structures in the cytosol to the plasma membrane and to the apex of intracellular and extracellular tachyzoites. While the enzyme shows preferential substrate specificity toward PtdIns over PtdIns(4,5)P_2_ *in vitro* (Fang et al., 2006), the most likely *in vivo* substrate is PtdIns(4,5)P_2_. That an extracellular drop in K^+^ levels activates TgPI-PLC-dependent intracellular Ca^2+^ mobilization reveals similar regulatory mechanisms with *Plasmodium* PI-PLC isoforms (Figure 5C) (Bullen et al., 2016).

*P. falciparum* PKG-mediated sensing of cGMP (PF3D7_1436600) is associated with Ca^2+^ mobilization-dependent processes, e.g., inhibition of PfPKG disrupted exoneme and microneme release and compromised egress of merozoites (Collins et al., 2013). Moreover, in *P. berghei*, a putative PKG (PBANKA_1008200) controls activation of PbPI4KIIIβ and peripheral localization of PbPI4P5K via phosphorylation of these enzymes in ookinetes. Additionally, PKG activity is critical for maintaining high cytosolic Ca^2+^ levels in gliding ookinetes. There is a direct link between PKG, PIP metabolism, and Ca^2+^ signaling as evidenced by the demonstration that PKG activation leads to rapid PtdIns(4,5)P_2_ hydrolysis and elevations in intracellular Ca^2+^ in *P. falciparum* schizonts. This is consistent with a role for PKG in regulating Ca^2+^ mobilization-dependent processes during this life cycle stage. In gliding ookinetes (where Ca^2+^ levels are high), PKG inhibition elicits the opposite effect. PtdIns levels increase while PIP and PtdIns(4,5)P_2_ levels decrease. These data suggest that PKG regulates the rate-limiting conversion of PtdIns to PtdIns4P in the ookinete stage via phosphorylation of PI4KIIIβ (Brochet et al., 2014).

In *T. gondii*, increases in intracellular Ca^2+^ can trigger the microneme discharge required for parasite invasion, motility, and egress (Carruthers and Sibley, 1999; Lourido et al., 2012). *T. gondii* encode multiple PKG isoforms and only isoform I (TGME49_311360) is essential for parasite invasion, motility, and for triggering microneme secretion (Wiersma et al., 2004; Brown et al., 2017). As in *Plasmodium* PKG, TgPKG isoform I-mediated sensing of cGMP induces Ca^2+^ release. Nonetheless, serum albumin-dependent microneme release, which is mediated by TgPKG activation, does not require Ca^2+^ mobilization (Brown et al., 2016). Although TgPKG and its direct involvement with PtdIns(4,5)P_2_ metabolism has not been addressed, it is suggested that TgPKG-dependent Ca^2+^ mobilization occurs upon upregulation of PtdIns4P and PtdIns(4,5)P_2_ synthesis, as shown in *P. falciparum* schizonts (Brochet et al., 2014; Bisio and Soldati-Favre, 2019). In addition, it is possible that an alternate pathway for stimulation of microneme secretion regulated by TgPKG that does not require intracellular Ca^2+^ mobilization and is triggered by serum albumin exists (Brown et al., 2016).

### Inositol phosphates (IPs) in Apicomplexa

IPs are highly polar and energy-rich molecule species that regulate important cellular signaling functions in eukaryotes (Dong et al., 2019; Sahu et al., 2020). IP_3_ also serves as a precursor of inositol hexakisphosphate (IP_6_) and its inositol pyrophosphates derivatives PP-IP_5_ (or IP_7_) and (PP)_2_-IP_4_ (or IP_8_) (Michell, 2008). A phylogenetic study detected the presence of genes coding for two out of the four eukaryotic kinase families involved in IP_3_ phosphorylation and of its derivatives in *P. falciparum* and *C. parvum,* the inositol polyphosphate kinase (IPK) and diphosphoinositol pentakisphosphate kinase (PPIP5K) (Laha et al., 2021), respectively. This finding suggests limited production of inositol pyrophosphates in Apicomplexans.

In Apicomplexa, the IP_3_ content has been detected and measured after addressing its involvement in the activation of Ca^2+^ mobilization following rapid PIP_2_ hydrolysis in *Plasmodium* (Raabe A. C. et al., 2011). Interestingly, genes encoding members of the IP_3_ receptor (IP_3_R) family are absent in this phylum (Naganume et al., 2006; Ladenburger et al., 2009). However, addition of IP_3_ and known IP_3_R competitive inhibitors stimulate and decrease Ca^2+^ release in *T. gondii in vitro*, respectively (Chini et al., 2005). Moreover, IP_3_ production is reduced following pharmacological inhibition of ryanodine receptor (RyR)-like channels, suggesting that RyR-mediated Ca^2+^ release stimulates sustained PbPI-PLCδ activity (Raabe A. C. et al., 2011). These results indicate that Apicomplexans have unique IP_3_R-like receptors with similar regulation instructions described in mammalian cells (Berridge et al., 2003).

## PtdIns5P in Apicomplexa

PtdIns5P in metazoa is proposed to be involved in cytoskeleton organization, modulation of transcriptional activity, T cell signaling, and regulation of endosomal protein sorting and autophagocytotic events. However, its metabolism and cellular functions remain poorly understood (Niebuhr, et al., 2002; Gozani et al., 2003; Guittard et al., 2009; Boal et al., 2015; Vicinanza et al., 2015). PtdIns5P is produced in multicellular organisms and in wild-type *S. cerevisiae* at relatively small levels (Zolov et al., 2012). The phylogenetic distribution of proteins implicated in the regulation of PtdIns5P metabolism and of its precursors in eukaryotes reveals four groups of co-evolving proteins (Lecompte et al., 2008). These groups (or triads) comprise a phosphatase, a kinase, and a regulator. Examples include the following: 1) the Vps15p–class III PI3K—enzymatically active MTM (aMTM) complex that regulates PtdIns3P turnover; 2) the Vac14—PIKfyve—Figure 4 complex that regulates the balance between PtdIns3P and PtdIns(3,5)P_2_; 3) the class I PI3K—catalytically dead MTM (dMTM)–aMTM complex initially hypothesized to regulate the interconversion between PtdIns(3,5)P_2_ and PtdIns5P, and 4) the PI4P5KII–PtdIns(4,5)P_2_ 4-phosphatase complex that converts PtdIns5P to PtdIns(4,5)P_2_ (and *vice versa*) found exclusively in Metazoa (Figure 5A) (Lecompte et al., 2008). PIKfyve is the major activity for producing PtdIns5P from PtdIns in mammals (Sbrissa et al., 2012; Zolov et al., 2012; Shisheva, 2013).

As reviewed in previous sections, some components from these complexes are either poorly conserved in *T. gondii* and *Plasmodium* (PIK3R4–PIK3C3–aMTM and VAC14–PIKfyve–FIG4) or not conserved at all (PIK3C–dMTM–aMTM and PIP5K2–PtdIns(4,5)P_2_ 4-phosphatases) (Supplementary Table S1). Nonetheless, PtdIns5P is inferred to be present in *P. falciparum* on the basis of membrane recruitment of the PtdIns5P-binding PH domain of mammalian PHDOK5 (downstream of tyrosine kinase 5). By this criterion, PtdIns5P is present in the plasma membrane and in the cytoplasm in the early stages of the intraerythrocytic cycle (Figure 2A). During trophozoite maturation, PtdIns5P is transiently detected in the nucleus (Figure 5B). During the schizont stage, PtdIns5P adopts a profile similar to that of PfSec13p (a marker of the transitional ER) (Ebrahimzadeh et al., 2018). These localization data suggest that the functions and regulation of PtdIns5P in *P. falciparum* and other Apicomplexans diverge from those of other eukaryotic organisms studied thus far.

## Regulation of host PI3K by Apicomplexan organisms

Apicomplexan parasites modulate host cell physiological processes to persist in host cells. One such strategy involves manipulation of host PI3K class I activation at the host cell plasma membrane to promote parasite invasion. *C. parvum* induces the accumulation and activation of host PI3K class IA at the host cell–parasite interface during invasion of biliary epithelial cells (Chen et al., 2004). Activation of host PI3K class IA leads to host cell actin remodeling at the host cell–parasite interface that potentiates the invasion process. How *C. parvum* activates PI3K in this process remains unclear. A potential mechanism involves *C. parvum* host cell attachment-dependent phosphorylation of host PI3K class IA. This hypothesis is supported by elevated tyrosine phosphorylation of the host PI3K p85 subunit in *C. parvum*-infected cells. Such PI3K class IA activation is expected to nucleate Cdc42 accumulation and activation at the host cell–parasite interface with subsequent actin remodeling at that site (Chen et al., 2004).

Strategies that control host cell survival upon parasite infection are also linked to parasite-mediated host PI3K activation. *P. berghei* induces early release of hepatocyte growth factor (HGF) during sporozoite invasion (Leirião et al., 2005). Interaction of HGF with its receptor c-MET promotes survival of uninfected hepatocytes (Carrolo et al., 2003). This interaction is amplified by sporozoite migration through the hepatocyte, and this process induces cell wounding and enhanced release of HGF. HGF/MET signaling produces an antiapoptotic response at early time points of infection via activation of the PI3-kinase/Akt pathway (Leirião et al., 2005). *Theileria parva*, the causative agent of East Coast fever, a particularly problematic disease of cattle in Africa, also promotes host cell immortalization that requires irreversible activation of class I PI3K. *T. parva*-transformed B cells further augment their proliferative potential via a granulocyte–monocyte colony-stimulating factor (GM-CSF) autocrine loop that sustains host class I PI3K activity and thereby stimulates activation of the transcription factor AP-1 (Activator protein 1) (Baumgartner et al., 2000; Dessauge et al., 2005).

Lastly, an important strategy that enables Apicomplexans to invade and survive within the host cell environment is the ability to evade destruction by the host. This capability is well-documented for *T. gondii* and involves activation of the PI3K/AKT pathway. During infection, *T. gondii* releases excreted/secreted antigens (TgESAs) that interact with cell surface receptors of macrophages and dendritic cells and thereby modulate the immune response (Wang et al., 2017). Such upregulation of the host PI3K/AKT pathway by TgESAs results in adverse pregnancy outcomes in mice (Chen et al., 2019), and also results in negative modulation of Forkhead box-p3 (Foxp3) expression. Foxp3 is a transcription factor whose function is critical for activation of CD4^+^CD25^+^ regulatory T cell (Treg) differentiation (So and Croft, 2007). The result of functional suppression of Fox3p is insufficiency of the Treg response to *T. gondii* infection. Moreover, TgESA-treated dendritic cells show elevated PI3K/AKT pathway activity that leads to diminished production of exogenous and endogenous reactive oxygen species (ROS). Downregulation of ROS production creates a host environment that favors *T. gondii* proliferation (Choi et al., 2020). Among the TgESAs, microneme proteins 3 and 6 (MIC3 and MIC6) are epidermal growth factor receptor ligands that activate the PI3K/Akt signaling pathway and dampen autophagy protein LC3 expression and parasitophorous vacuole–lysosomal fusion. This circuit protects the parasite from clearance by the host autophagy machinery (Muniz-Feliciano et al., 2013; Wang et al., 2016; Zhu et al., 2019). These examples show that Apicomplexans not only have machinery to regulate their endogenous PIP levels but have also developed the means for regulating host PIP levels involved in signaling pathways that activate/inactivate host responses to the benefit of the parasite.

## Concluding remarks

Studies focused on PIP metabolism and its regulation in Apicomplexa are in their infancy. Among the 17 prospective PtdIns and PIP kinase classes identified in *Plasmodium* and *T. gondii*, only five have been characterized in detail (PfPI3K, TgPI3K, PfPI4KIIIβ, PfPI4P5K, and TgPIKfyve). Reports of PIP phosphatases and regulatory factors that mediate PdtIns/PIP synthesis and catabolism in Apicomplexa are similarly sparse. This lack of information is reflected in the relative lack of quality small-molecule inhibitors that target PI/PIP signaling pathways, especially in *T. gondii* and other members of the phylum. This would seem a fruitful area for future research given that a detailed understanding of the shape of the apicomplexan PIP signaling landscape holds the potential for interesting comparisons regarding how this major intracellular signaling system evolved in these unusual parasites relative to its adaptation to multiple regulatory roles in higher eukaryotes.

The presence and compartmental distribution of most PIP species has been mainly addressed in the intraerythrocytic stages of *P. falciparum* parasites. Few PIP species have been detected and mapped in *T. gondii* or other members of the phylum, however. Expansion of PIP studies to Apicomplexa with life cycles diverged from those of *Plasmodium* will further inform how the built-in plasticity of PIP signaling networks is suited for regulation of complex developmental differences in evolutionarily related organisms.

Synthesis and spatial arrangement of PIPs are tightly controlled during division in higher eukaryotic cells and yeast (Schorr et al., 2001; Cauvin and Echard, 2015). PIP synthesis regulation throughout the cell cycle in specific life cycle stages has been poorly explored in Apicomplexa. Complexities of this phylum, two-host life cycle, unconventional cell division phases, and remarkably fast multiplication–make Apicomplexa an ideal model to understand how eukaryotes can adjust or recycle PIPs during progression of cell division.

Factors that regulate Apicomplexan PIP signaling are often found by virtue of their orthology to mammalian/yeast PdtIns/PIP kinase regulators. Several of these factors are seemingly absent in *Plasmodium* and *T. gondii*. Expansion on studies addressing uncharacterized Apicomplexan-specific proteins would inform fundamental aspects of how PIP production is regulated in complex unicellular and multicellular eukaryotes.

Finally, PIP pathways in single-celled eukaryotes have proven potent and specific drug targets, e.g., the imidazopyrazine compounds that target *Plasmodium* PI4KIIIβ activity or multiple small-molecule inhibitors of Sec14 in yeast (McNamara et al., 2013; Nile et al., 2014; Filipuzzi et al., 2016; Khan et al., 2021). In this review, we listed undescribed essential orthologs of mammalian and yeast proteins involved in regulation of PIP synthesis in *Plasmodium* and *T. gondii*. Characterization of these proteins, specifically the ones that diverge from their counterparts in higher organisms, promises to reveal effective strategies for designing antiparasitic drugs with high target selectivities.

## References

[B1] Abrahamsen M. S. Templeton T. J. Enomoto S. Abrahante J. E. Zhu G. Lancto C. A. (2004). Complete genome sequence of the Apicomplexan, Cryptosporidium parvum. Science 304 (5669), 441–445. 10.1126/science.1094786 15044751

[B2] Ackers J. P. (1997). Gut coccidia-isospora, Cryptosporidium, Cyclospora and Sarcocystis. Seminars Gastrointest. Dis. 8 (No. 1), 33–44. 9000500

[B3] Al-Khedery B. Barnwell J. W. Galinski M. R. (1999). Stage-specific expression of 14-3-3 in asexual blood-stage Plasmodium. Mol. Biochem. Parasitol. 102, 117–130. 10.1016/s0166-6851(99)00090-0 10477181

[B4] Arabiotorre A. Formanowicz M. Bankaitis V. A. Grabon A. (2023). Phosphatidylinositol-4-phosphate signaling regulates dense granule biogenesis and exocytosis in Toxoplasma gondii, 1–46. bioRxiv. 10.1371/journal.ppat.1014451 PMC13472501 42497226

[B5] Ariey F. Witkowski B. Amaratunga C. Beghain J. Langlois A. C. Khim N. (2014). A molecular marker of artemisinin-resistant Plasmodium falciparum malaria. Nature 505 (7481), 50–55. 10.1038/nature12876 24352242 PMC5007947

[B6] Astle M. V. Seaton G. Davies E. M. Fedele C. G. Rahman P. Arsala L. (2006). Regulation of phosphoinositide signaling by the inositol poly phosphate 5-phosphatases. IUBMB Life 58, 451–456. 10.1080/15216540600871159 16916781

[B7] Audhya A. Foti M. Emr S. D. (2000). Distinct roles for the yeast phosphatidylinositol 4-kinases, Stt4p and Pik1p, in secretion, cell growth, and organelle membrane dynamics. Mol. Biol. Cell 11 (8), 2673–2689. 10.1091/mbc.11.8.2673 10930462 PMC14948

[B8] Bai M. J. Wang J. L. Elsheikha H. M. Liang Q. L. Chen K. Nie L. B. (2018). Functional characterization of dense granule proteins in Toxoplasma gondii RH strain using CRISPR-Cas9 system. Front. Cell. Infect. Microbiol. 8, 300. 10.3389/fcimb.2018.00300 30211128 PMC6121064

[B9] Balla A. Tuymetova G. Barshishat M. Geiszt M. Balla T. (2002). Characterization of type II phosphatidylinositol 4-kinase isoforms reveals association of the enzymes with endosomal vesicular compartments. J. Biol. Chem. 277 (22), 20041–20050. 10.1074/jbc.M111807200 11923287

[B10] Balla T. (2013). Phosphoinositides: tiny lipids with giant impact on cell regulation. Physiol. Rev. 93 (3), 1019–1137. 10.1152/physrev.00028.2012 23899561 PMC3962547

[B11] Bankaitis V. A. Malehorn D. E. Emr S. D. Greene R. (1989). The *Saccharomyces cerevisiae* SEC14 gene encodes a cytosolic factor that is required for transport of secretory proteins from the yeast Golgi complex. J. Cell Biol. 108 (4), 1271–1281. 10.1083/jcb.108.4.1271 2466847 PMC2115512

[B12] Bankaitis V. A. Mousley C. J. Schaaf G. (2010). The Sec14 superfamily and mechanisms for crosstalk between lipid metabolism and lipid signaling. Trends Biochem. Sci. 35 (3), 150–160. 10.1016/j.tibs.2009.10.008 19926291 PMC2834860

[B13] Bansal P. Antil N. Kumar M. Yamaryo-Botté Y. Rawat R. S. Pinto S. (2021). Protein kinase TgCDPK7 regulates vesicular trafficking and phospholipid synthesis in Toxoplasma gondii. PLoS Pathog. 17 (2), e1009325. 10.1371/journal.ppat.1009325 33635921 PMC7909640

[B14] Bansal P. Tripathi A. Thakur V. Mohmmed A. Sharma P. (2017). Autophagy-related protein ATG18 regulates apicoplast biogenesis in Apicomplexan parasites. MBio 8 (5), 1–16. 10.1128/mBio.01468-17 PMC5666157 29089429

[B15] Batrouni A. G. Baskin J. M. (2021). The chemistry and biology of phosphatidylinositol 4-phosphate at the plasma membrane. Bioorg. Med. Chem. 40, 116190. 10.1016/j.bmc.2021.116190 33965837 PMC8169619

[B16] Baumgartner M. Chaussepied M. Moreau M. F. Werling D. Davis W. C. Garcia A. (2000). Constitutive PI3‐K activity is essential for proliferation, but not survival, of Theileria parva‐transformed B cells. Cell. Microbiol. 2 (4), 329–339. 10.1046/j.1462-5822.2000.00062.x 11207589

[B17] Bergmann A. Floyd K. Key M. Dameron C. Rees K. C. Thornton L. B. (2020). Toxoplasma gondii requires its plant-like heme biosynthesis pathway for infection. PLoS pathogens 16 (5), 1008499. 10.1371/journal.ppat.1008499 PMC7252677 32407406

[B18] Berridge M. J. Bootman M. D. Roderick H. L. (2003). Calcium signalling: dynamics, homeostasis and remodelling. Nat. Rev. Mol. Cell Biol. 4 (7), 517–529. 10.1038/nrm1155 12838335

[B19] Berridge M. J. Irvine R. F. (1984). Inositol trisphosphate, a novel second messenger in cellular signal transduction. Nature 312 (5992), 315–321. 10.1038/312315a0 6095092

[B20] Besteiro S. (2017). Autophagy in apicomplexan parasites. Curr. Opin. Microbiol. 40, 14–20. 10.1016/j.mib.2017.10.008 29096193

[B21] Bisio H. Soldati-Favre D. (2019). Signaling cascades governing entry into and exit from host cells by Toxoplasma gondii. Annu. Rev. Microbiol. 73, 579–599. 10.1146/annurev-micro-020518-120235 31500539

[B22] Blagoveshchenskaya A. Cheong F. Y. Rohde H. M. Glover G. Knodler A. Nicolson T. (2008). Integration of Golgi trafficking and growth factor signaling by the lipid phosphatase SAC1. J. Cell Biol. 180 (4), 803–812. 10.1083/jcb.200708109 18299350 PMC2265582

[B23] Boal F. Mansour R. Gayral M. Saland E. Chicanne G. Xuereb J. M. (2015). TOM1 is a PI5P effector involved in the regulation of endosomal maturation. J. Cell Sci. 128 (4), 815–827. 10.1242/jcs.166314 25588840

[B24] Botelho R. J. Efe J. A. Teis D. Emr S. D. (2008). Assembly of a Fab1 phosphoinositide kinase signaling complex requires the Fig4 phosphoinositide phosphatase. Mol. Biol. Cell 19 (10), 4273–4286. 10.1091/mbc.e08-04-0405 18653468 PMC2555960

[B25] Brearley C. A. Hanke D. E. (1993). Pathway of synthesis of 3, 4-and 4, 5-phosphorylated phosphatidylinositols in the duckweed Spirodela polyrhiza L. Biochem. J. 290 (1), 145–150. 10.1042/bj2900145 8382475 PMC1132394

[B26] Brochet M. Collins M. O. Smith T. K. Thompson E. Sebastian S. Volkmann K. (2014). Phosphoinositide metabolism links cGMP-dependent protein kinase G to essential Ca²⁺ signals at key decision points in the life cycle of malaria parasites. PLoS Biol. 12 (3), e1001806. 10.1371/journal.pbio.1001806 24594931 PMC3942320

[B27] Brown J. R. Auger K. R. (2011). Phylogenomics of phosphoinositide lipid kinases: perspectives on the evolution of second messenger signaling and drug discovery. BMC Evol. Biol. 11 (1), 4–14. 10.1186/1471-2148-11-4 21208444 PMC3024228

[B28] Brown K. M. Long S. Sibley L. D. (2017). Plasma membrane association by N-acylation governs PKG function in Toxoplasma gondii. MBio 8 (3), 1–14. 10.1128/mBio.00375-17 PMC5414004 28465425

[B29] Brown K. M. Lourido S. Sibley L. D. (2016). Serum albumin stimulates protein kinase G-dependent microneme secretion in Toxoplasma gondii. J. Biol. Chem. 291 (18), 9554–9565. 10.1074/jbc.M115.700518 26933037 PMC4850294

[B30] Bullen H. E. Jia Y. Yamaryo-Botté Y. Bisio H. Zhang O. Jemelin N. K. (2016). Phosphatidic acid-mediated signaling regulates microneme secretion in Toxoplasma. Cell host microbe 19 (3), 349–360. 10.1016/j.chom.2016.02.006 26962945

[B31] Bunney T. D. Katan M. (2011). PLC regulation: emerging pictures for molecular mechanisms. Trends Biochem. Sci. 36 (2), 88–96. 10.1016/j.tibs.2010.08.003 20870410

[B32] Burke J. E. Inglis A. J. Perisic O. Masson G. R. McLaughlin S. H. Rutaganira F. (2014). Structures of PI4KIIIβ complexes show simultaneous recruitment of Rab11 and its effectors. Science 344 (6187), 1035–1038. 10.1126/science.1253397 24876499 PMC4046302

[B33] Burke J. E. (2018). Structural basis for regulation of phosphoinositide kinases and their involvement in human disease. Mol. Cell 71 (5), 653–673. 10.1016/j.molcel.2018.08.005 30193094

[B34] Bushell E. Gomes A. R. Sanderson T. Anar B. Girling G. Herd C. (2017). Functional profiling of a Plasmodium genome reveals an abundance of essential genes. Cell 170 (2), 260–272.e8. 10.1016/j.cell.2017.06.030 28708996 PMC5509546

[B35] Cantley L. C. (2002). The phosphoinositide 3-kinase pathway. Science 296 (5573), 1655–1657. 10.1126/science.296.5573.1655 12040186

[B36] Carrolo M. Giordano S. Cabrita-Santos L. Corso S. Vigário A. M. Silva S. (2003). Hepatocyte growth factor and its receptor are required for malaria infection. Nat. Med. 9 (11), 1363–1369. 10.1038/nm947 14556002

[B37] Carruthers V. B. Sibley L. D. (1999). Mobilization of intracellular calcium stimulates microneme discharge in Toxoplasma gondii. Mol. Microbiol. 31 (2), 421–428. 10.1046/j.1365-2958.1999.01174.x 10027960

[B38] Cauvin C. Echard A. (2015). Phosphoinositides: lipids with informative heads and mastermind functions in cell division. Biochimica Biophysica Acta (BBA)-Molecular Cell Biol. Lipids 1851 (6), 832–843. 10.1016/j.bbalip.2014.10.013 25449648

[B39] Cernikova L. Faso C. Hehl A. B. (2019). Roles of phosphoinositides and their binding proteins in parasitic protozoa. Trends Parasitol. 35 (12), 996–1008. 10.1016/j.pt.2019.08.008 31615721

[B40] Cerqueira G. C. Cheeseman I. H. Schaffner S. F. Nair S. McDew-White M. Phyo A. P. (2017). Longitudinal genomic surveillance of Plasmodium falciparum malaria parasites reveals complex genomic architecture of emerging artemisinin resistance. Genome Biol. 18 (1), 78–13. 10.1186/s13059-017-1204-4 28454557 PMC5410087

[B41] Chakraborty S. Roy S. Mistry H. U. Murthy S. George N. Bhandari V. (2017). Potential sabotage of host cell physiology by Apicomplexan parasites for their survival benefits. Front. Immunol. 8, 1261. 10.3389/fimmu.2017.01261 29081773 PMC5645534

[B42] Chang F. S. Han G. S. Carman G. M. Blumer K. J. (2005). A WASp-binding type II phosphatidylinositol 4-kinase required for actin polymerization-driven endosome motility. J. Cell Biol. 171 (1), 133–142. 10.1083/jcb.200501086 16216926 PMC2171216

[B43] Chen J. Hu L. Wang J. Cao Y. Zhu D. Chen L. (2019). Toxoplasma gondii excreted‐secreted antigens suppress Foxp3 via PI3K‐AKT‐mTOR signaling pathway. J. Cell. Biochem. 120 (9), 16044–16051. 10.1002/jcb.28884 31074049

[B44] Chen X. M. Splinter P. L. Tietz P. S. Huang B. Q. Billadeau D. D. LaRusso N. F. (2004). Phosphatidylinositol 3-kinase and frabin mediate Cryptosporidium parvum cellular invasion via activation of Cdc42. J. Biol. Chem. 279 (30), 31671–31678. 10.1074/jbc.M401592200 15133042

[B45] Chini E. N. Nagamune K. Wetzel D. M. Sibley L. D. (2005). Evidence that the cADPR signalling pathway controls calcium-mediated microneme secretion in Toxoplasma gondii. Biochem. J. 389 (2), 269–277. 10.1042/BJ20041971 15773818 PMC1175103

[B46] Choi H. G. Gao F. F. Zhou W. Sun P. R. Yuk J. M. Lee Y. H. (2020). The role of PI3K/AKT pathway and NADPH oxidase 4 in host ROS manipulation by Toxoplasma gondii. Korean J. Parasitol. 58 (3), 237–247. 10.3347/kjp.2020.58.3.237 32615737 PMC7338895

[B47] Chung J. Torta F. Masai K. Lucast L. Czapla H. Tanner L. B. (2015). INTRACELLULAR TRANSPORT. PI4P/phosphatidylserine countertransport at ORP5- and ORP8-mediated ER-plasma membrane contacts. Science 349 (6246), 428–432. 10.1126/science.aab1370 26206935 PMC4638224

[B48] Clayton E. L. Minogue S. Waugh M. G. (2013). Phosphatidylinositol 4-kinases and PI4P metabolism in the nervous system: roles in psychiatric and neurological diseases. Mol. Neurobiol. 47 (1), 361–372. 10.1007/s12035-012-8358-6 23054682

[B49] Collins C. R. Hackett F. Strath M. Penzo M. Withers-Martinez C. Baker D. A. (2013). Malaria parasite cGMP-dependent protein kinase regulates blood stage merozoite secretory organelle discharge and egress. PLoS Pathog. 9 (5), e1003344. 10.1371/journal.ppat.1003344 23675297 PMC3649973

[B50] Coppens I. Asai T. Tomavo S. (2014). “Biochemistry and metabolism of Toxoplasma gondii: carbohydrates, lipids and nucleotides,” in Toxoplasma gondii (Cambridge: Academic Press), 257–295.

[B51] Coppens I. Sinai A. P. Joiner K. A. (2000). Toxoplasma gondii exploits host low-density lipoprotein receptor-mediated endocytosis for cholesterol acquisition. J. Cell Biol. 149 (1), 167–180. 10.1083/jcb.149.1.167 10747095 PMC2175092

[B52] Cunningham T. W. Lips D. L. Bansal V. S. Caldwell K. K. Mitchell C. A. Majerus P. W. (1990). Pathway for the formation of D-3 phosphate containing inositol phospholipids in intact human platelets. J. Biol. Chem. 265 (35), 21676–21683. 10.1016/s0021-9258(18)45793-6 2174884

[B53] Daboussi L. Costaguta G. Ghukasyan R. Payne G. S. (2017). Conserved role for Gga proteins in phosphatidylinositol 4-kinase localization to the trans-Golgi network. Proc. Natl. Acad. Sci. 114 (13), 3433–3438. 10.1073/pnas.1615163114 28289207 PMC5380026

[B54] Daher W. Morlon-Guyot J. Alayi T. D. Tomavo S. Wengelnik K. Lebrun M. (2016). Identification of Toxoplasma TgPH1, a pleckstrin homology domain-containing protein that binds to the phosphoinositide PI(3,5)P2. Mol. Biochem. Parasitol. 207 (1), 39–44. 10.1016/j.molbiopara.2016.03.011 27063980

[B55] Daher W. Morlon‐Guyot J. Sheiner L. Lentini G. Berry L. Tawk L. (2015). Lipid kinases are essential for apicoplast homeostasis in Toxoplasma gondii. Cell. Microbiol. 17 (4), 559–578. 10.1111/cmi.12383 25329540 PMC4356647

[B56] D’Angelo G. Polishchuk E. Tullio G. D. Santoro M. Campli A. D. Godi A. (2007). Glycosphingolipid synthesis requires FAPP2 transfer of glucosylceramide. Nature 449 (7158), 62–67. 10.1038/nature06097 17687330

[B57] De Craene J. O. Bertazzi D. L. Bär S. Friant S. (2017). Phosphoinositides, major actors in membrane trafficking and lipid signaling pathways. Int. J. Mol. Sci. 18 (3), 634. 10.3390/ijms18030634 28294977 PMC5372647

[B58] Del Bel L. M. Brill J. A. (2018). Sac1, a lipid phosphatase at the interface of vesicular and nonvesicular transport. Traffic 19 (5), 301–318. 10.1111/tra.12554 29411923

[B59] del Mar Siles-Lucas M. Gottstein B. (2003). The 14-3-3 protein: a key molecule in parasites as in other organisms. Trends Parasitol. 19 (12), 575–581. 10.1016/j.pt.2003.10.003 14642768

[B60] Desrivières S. Cooke F. T. Parker P. J. Hall M. N. (1998). MSS4, a phosphatidylinositol-4-phosphate 5-kinase required for organization of the actin cytoskeleton in *Saccharomyces cerevisiae* . J. Biol. Chem. 273 (25), 15787–15793. 10.1074/jbc.273.25.15787 9624178

[B61] Dessauge F. Lizundia R. Langsley G. (2005). Constitutively activated CK2 potentially plays a pivotal role in Theileria-induced lymphocyte transformation. Parasitology 130 (S1), S37–S44. 10.1017/S0031182005008140 16281991

[B62] Di Paolo G. De Camilli P. (2006). Phosphoinositides in cell regulation and membrane dynamics. Nature 443 (7112), 651–657. 10.1038/nature05185 17035995

[B63] Dickson E. J. Jensen J. B. Hille B. (2014). Golgi and plasma membrane pools of PI(4)P contribute to plasma membrane PI(4,5)P2 and maintenance of KCNQ2/3 ion channel current. Proc. Natl. Acad. Sci. 111 (22), E2281–E2290. 10.1073/pnas.1407133111 24843134 PMC4050574

[B64] Divecha N. Roefs M. Halstead J. R. D'Andrea S. Fernandez-Borga M. Oomen L. (2000). Interaction of the type ialpha PIPkinase with phospholipase D: a role for the local generation of phosphatidylinositol 4, 5-bisphosphate in the regulation of PLD2 activity. EMBO J. 19 (20), 5440–5449. 10.1093/emboj/19.20.5440 11032811 PMC314009

[B65] Dluzewski A. R. Ling I. T. Hopkins J. M. Grainger M. Margos G. Mitchell G. H. (2008). Formation of the food vacuole in Plasmodium falciparum: a potential role for the 19 kDa fragment of merozoite surface protein 1 (MSP119). PloS *one* . PLoS One 3 (8), e3085. 10.1371/journal.pone.0003085 18769730 PMC2518119

[B66] Dong J. Ma G. Sui L. Wei M. Satheesh V. Zhang R. (2019). Inositol pyrophosphate InsP8 acts as an intracellular phosphate signal in Arabidopsis. Mol. plant 12 (11), 1463–1473. 10.1016/j.molp.2019.08.002 31419530

[B67] Duex J. E. Nau J. J. Kauffman E. J. Weisman L. S. (2006). Phosphoinositide 5-phosphatase Fig4p is required for both acute rise and subsequent fall in stress-induced phosphatidylinositol 3, 5-bisphosphate levels. Eukaryot. Cell 5 (4), 723–731. 10.1128/EC.5.4.723-731.2006 16607019 PMC1459661

[B68] Ebrahimzadeh Z. Mukherjee A. Richard D. (2018). A map of the subcellular distribution of phosphoinositides in the erythrocytic cycle of the malaria parasite Plasmodium falciparum. Int. J. Parasitol. 48 (1), 13–25. 10.1016/j.ijpara.2017.08.015 29154995

[B69] Fan W. Nassiri A. Zhong Q. (2011). Autophagosome targeting and membrane curvature sensing by Barkor/Atg14 (L). Proc. Natl. Acad. Sci. 108 (19), 7769–7774. 10.1073/pnas.1016472108 21518905 PMC3093500

[B70] Fang J. Marchesini N. Moreno S. N. (2006). A Toxoplasma gondii phosphoinositide phospholipase C (TgPI-PLC) with high affinity for phosphatidylinositol. Biochem. J. 394 (2), 417–425. 10.1042/BJ20051393 16288600 PMC1408672

[B71] Fang M. Kearns B. G. Gedvilaite A. Kagiwada S. Kearns M. Fung M. K. (1996). Kes1p shares homology with human oxysterol binding protein and participates in a novel regulatory pathway for yeast Golgi‐derived transport vesicle biogenesis. EMBO J. 15 (23), 6447–6459. 10.1002/j.1460-2075.1996.tb01036.x 8978672 PMC452470

[B72] Ferguson D. J. P. Hutchison W. M. Dunachie J. F. Siim J. C. (1974). Ultrastructural study of early stages of asexual multiplication and microgametogony of Toxoplasma gondii in the small intestine of the cat. Acta Pathologica Microbiol. Scand. Sect. B Microbiol. Immunol. 82 (2), 167–181. 10.1111/j.1699-0463.1974.tb02309.x 4528099

[B73] Filipuzzi I. Cotesta S. Perruccio F. Knapp B. Fu Y. Studer C. (2016). High-resolution genetics identifies the lipid transfer protein Sec14p as target for antifungal ergolines. PLoS Genet. 12 (11), e1006374. 10.1371/journal.pgen.1006374 27855158 PMC5147771

[B74] Francia M. E. Striepen B. (2014). Cell division in Apicomplexan parasites. Nat. Rev. Microbiol. 12 (2), 125–136. 10.1038/nrmicro3184 24384598

[B75] Fu J. Zhao L. Pang Y. Chen H. Yamamoto H. Chen Y. (2022). Apicoplast biogenesis mediated by ATG8 requires the ATG12–ATG5-ATG16L and SNAP29 complexes in Toxoplasma gondii. Autophagy 19 (4), 1258–1276. 10.1080/15548627.2022.2123639 36095096 PMC10012919

[B76] Garcia P. Gupta R. Shah S. Morris A. J. Rudge S. A. Scarlata S. (1995). The pleckstrin homology domain of phospholipase C-delta 1 binds with high affinity to phosphatidylinositol 4,5-bisphosphate in bilayer membranes. Biochemistry 34 (49), 16228–16234. 10.1021/bi00049a039 8519781

[B77] Gary J. D. Sato T. K. Stefan C. J. Bonangelino C. J. Weisman L. S. Emr S. D. (2002). Regulation of Fab1 phosphatidylinositol 3-phosphate 5-kinase pathway by Vac7 protein and Fig4, a polyphosphoinositide phosphatase family member. Mol. Biol. Cell 13 (4), 1238–1251. 10.1091/mbc.01-10-0498 11950935 PMC102265

[B78] Ghosh D. Walton J. L. Roepe P. D. Sinai A. P. (2012). Autophagy is a cell death mechanism in Toxoplasma gondii. Cell. Microbiol. 14 (4), 589–607. 10.1111/j.1462-5822.2011.01745.x 22212386 PMC3302960

[B79] Gillooly D. J. Morrow I. C. Lindsay M. Gould R. Bryant N. J. Gaullier J. M. (2000). Localization of phosphatidylinositol 3‐phosphate in yeast and mammalian cells. EMBO J. 19 (17), 4577–4588. 10.1093/emboj/19.17.4577 10970851 PMC302054

[B80] Gnädig N. F. Stokes B. H. Edwards R. L. Kalantarov G. F. Heimsch K. C. Kuderjavy M. (2020). Insights into the intracellular localization, protein associations and artemisinin resistance properties of Plasmodium falciparum K13. PLoS Pathog. 16 (4), e1008482. 10.1371/journal.ppat.1008482 32310999 PMC7192513

[B81] Gozani O. Karuman P. Jones D. R. Ivanov D. Cha J. Lugovskoy A. A. (2003). The PHD finger of the chromatin-associated protein ING2 functions as a nuclear phosphoinositide receptor. Cell 114 (1), 99–111. 10.1016/s0092-8674(03)00480-x 12859901

[B82] Gras S. Jimenez-Ruiz E. Klinger C. M. Schneider K. Klingl A. Lemgruber L. (2019). An endocytic-secretory cycle participates in Toxoplasma gondii in motility. PLoS Biol. 17 (6), e3000060. 10.1371/journal.pbio.3000060 31233488 PMC6611640

[B83] Griffith M. B. Pearce C. S. Heaslip A. T. (2022). Dense granule biogenesis, secretion, and function in Toxoplasma gondii. J. Eukaryot. Microbiol. 69, e12904. 10.1111/jeu.12904 35302693 PMC9482668

[B84] Gubbels M. J. Duraisingh M. T. (2012). Evolution of Apicomplexan secretory organelles. Int. J. Parasitol. 42 (12), 1071–1081. 10.1016/j.ijpara.2012.09.009 23068912 PMC3583008

[B85] Guittard G. Gérard A. Dupuis-Coronas S. Tronchère H. Mortier E. Favre C. (2009). Cutting edge: Dok-1 and Dok-2 adaptor molecules are regulated by phosphatidylinositol 5-phosphate production in T cells. J. Immunol. 182 (7), 3974–3978. 10.4049/jimmunol.0804172 19299694

[B86] Guo S. Stolz L. E. Lemrow S. M. York J. D. (1999). SAC1-like domains of yeast SAC1, INP52, and INP53 and of human synaptojanin encode polyphosphoinositide phosphatases. J. Biol. Chem. 274 (19), 12990–12995. 10.1074/jbc.274.19.12990 10224048

[B87] Hama H. Schnieders E. A. Thorner J. Takemoto J. Y. DeWald D. B. (1999). Direct involvement of phosphatidylinositol 4-phosphate in secretion in the yeast *Saccharomyces cerevisiae* . J. Biol. Chem. 274 (48), 34294–34300. 10.1074/jbc.274.48.34294 10567405

[B88] Hammond G. R. Balla T. (2015). Polyphosphoinositide binding domains: key to inositol lipid biology. Biochimica Biophysica Acta (BBA)-Molecular Cell Biol. Lipids 1851 (6), 746–758. 10.1016/j.bbalip.2015.02.013 PMC4380703 25732852

[B89] Han G. S. Audhya A. Markley D. J. Emr S. D. Carman G. M. (2002). The *Saccharomyces cerevisiae* LSB6 gene encodes phosphatidylinositol 4-kinase activity. J. Biol. Chem. 277 (49), 47709–47718. 10.1074/jbc.M207996200 12361950

[B90] Harding C. R. Meissner M. (2014). The inner membrane complex through development of Toxoplasma gondii and Plasmodium. Cell. Microbiol. 16 (5), 632–641. 10.1111/cmi.12285 24612102 PMC4286798

[B91] Harper J. M. Huynh M. H. Coppens I. Parussini F. Moreno S. Carruthers V. B. (2006). A cleavable propeptide influences Toxoplasma infection by facilitating the trafficking and secretion of the TgMIC2–M2AP invasion complex. Mol. Biol. Cell 17 (10), 4551–4563. 10.1091/mbc.e06-01-0064 16914527 PMC1635346

[B92] Hasegawa J. Strunk B. S. Weisman L. S. (2017). PI5P and PI(3,5)P2: minor, but essential phosphoinositides. Cell Struct. Funct. 42, 49. 10.1247/csf.17003 28302928 PMC5846621

[B93] Hausser A. Link G. Hoene M. Russo C. Selchow O. Pfizenmaier K. (2006). Phospho-specific binding of 14-3-3 proteins to phosphatidylinositol 4-kinase III β protects from dephosphorylation and stabilizes lipid kinase activity. J. Cell Sci. 119 (17), 3613–3621. 10.1242/jcs.03104 16912074

[B94] Hausser A. Storz P. Märtens S. Link G. Toker A. Pfizenmaier K. (2005). Protein kinase D regulates vesicular transport by phosphorylating and activating phosphatidylinositol-4 kinase IIIbeta at the Golgi complex. Nat. Cell Biol. 7 (9), 880–886. 10.1038/ncb1289 16100512 PMC1458033

[B95] Herneisen A. L. Li Z. H. Chan A. W. Moreno S. N. Lourido S. (2022). Temporal and thermal profiling of the Toxoplasma proteome implicates parasite Protein Phosphatase 1 in the regulation of Ca2+-responsive pathways. Elife 11, e80336. 10.7554/eLife.80336 35976251 PMC9436416

[B96] Highland C. M. Fromme J. C. (2021). Arf1 directly recruits the Pik1-Frq1 PI4K complex to regulate the final stages of Golgi maturation. Mol. Biol. Cell 32 (10), 1064–1080. 10.1091/mbc.E21-02-0069 33788598 PMC8101487

[B97] Hill R. J. Ringel A. Knuepfer E. Moon R. W. Blackman M. J. van Ooij C. (2016). Regulation and essentiality of the StAR-related lipid transfer (START) domain-containing phospholipid transfer protein PFA0210c in malaria parasites. J. Biol. Chem. 291 (46), 24280–24292. 10.1074/jbc.M116.740506 27694132 PMC5104948

[B98] Hinchliffe K. A. Irvine R. F. (2010). “Type I phosphatidylinositol 4-phosphate 5- kinases (P14P 5-kinases),” in Handbook of cell signaling. Editors Bradshaw R. A. Dennis E. A. (Cambridge: Elsevier Academic Press), p1037–p1041.

[B99] Ijuin T. Takenawa T. (2003). SKIP negatively regulates insulin-induced GLUT4 translocation and membrane ruffle formation. Mol. Cell. Biol. 23 (4), 1209–1220. 10.1128/MCB.23.4.1209-1220.2003 12556481 PMC141139

[B100] Ikonomov O. C. Sbrissa D. Shisheva A. (2006). Localized PtdIns 3, 5-P2 synthesis to regulate early endosome dynamics and fusion. Am. J. Physiology-Cell Physiology 291 (2), C393–C404. 10.1152/ajpcell.00019.2006 16510848

[B101] Ishihara H. Shibasaki Y. Kizuki N. Wada T. Yazaki Y. Asano T. (1998). Type I phosphatidylinositol-4-phosphate 5-kinases: cloning of the third isoform and deletion/substitution analysis of members of this novel lipid kinase family. J. Biol. Chem. 273 (15), 8741–8748. 10.1074/jbc.273.15.8741 9535851

[B102] Jin N. Chow C. Y. Liu L. Zolov S. N. Bronson R. Davisson M. (2008). VAC14 nucleates a protein complex essential for the acute interconversion of PI3P and PI(3,5)P(2) in yeast and mouse. EMBO J. 27 (24), 3221–3234. 10.1038/emboj.2008.248 19037259 PMC2600653

[B103] Jones D. R. Sanjuan M. A. Mérida I. (2000). Type Ialpha phosphatidylinositol 4-phosphate 5-kinase is a putative target for increased intracellular phosphatidic acid. FEBS Lett. 476 (3), 160–165. 10.1016/s0014-5793(00)01702-6 10913605

[B104] Jović M. Kean M. J. Szentpetery Z. Polevoy G. Gingras A. C. Brill J. A. (2012). Two phosphatidylinositol 4-kinases control lysosomal delivery of the Gaucher disease enzyme, β-glucocerebrosidase. Mol. Biol. Cell 23 (8), 1533–1545. 10.1091/mbc.E11-06-0553 22337770 PMC3327330

[B105] Kerk D. Moorhead G. B. (2010). A phylogenetic survey of myotubularin genes of eukaryotes: distribution, protein structure, evolution, and gene expression. BMC Evol. Biol. 10 (1), 1–16. 10.1186/1471-2148-10-196 20576132 PMC2927912

[B106] Khan D. Nile A. H. Tripathi A. Bankaitis V. A. (2021). Emerging prospects for combating fungal infections by targeting phosphatidylinositol transfer proteins. Int. J. Mol. Sci. 22 (13), 6754. 10.3390/ijms22136754 34201733 PMC8269425

[B107] Kong P. Lehmann M. J. Helms J. B. Brouwers J. F. Gupta N. (2018). Lipid analysis of Eimeria sporozoites reveals exclusive phospholipids, a phylogenetic mosaic of endogenous synthesis, and a host-independent lifestyle. Cell Discov. 4 (1), 24–17. 10.1038/s41421-018-0023-4 29844921 PMC5964319

[B108] Kotomura N. Tsunemine S. Kuragano M. Asanuma T. Nakagawa H. Tanaka K. (2018). Sfh1, an essential component of the RSC chromatin remodeling complex, maintains genome integrity in fission yeast. Genes Cells 23 (9), 738–752. 10.1111/gtc.12629 30155942

[B109] Koyama T. Ohsawa T. Shimada S. Omata Y. Xuan X. Inoue N. (2001). A 14-3-3 protein homologue is expressed in feline enteroepithelial-stages of Toxoplasma gondii. Veterinary Parasitol. 96 (1), 65–74. 10.1016/s0304-4017(00)00424-6 11182236

[B110] Koyanagi M. Ono K. Suga H. Iwabe N. Miyata T. (1998). Phospholipase C cDNAs from sponge and hydra: antiquity of genes involved in the inositol phospholipid signaling pathway. FEBS Lett. 439 (1-2), 66–70. 10.1016/s0014-5793(98)01339-8 9849879

[B111] Ladenburger E. M. Sehring I. M. Korn I. Plattner H. (2009). Novel types of Ca2+ release channels participate in the secretory cycle of Paramecium cells. Mol. Cell. Biol. 29 (13), 3605–3622. 10.1128/MCB.01592-08 19380481 PMC2698757

[B112] Laha D. Portela-Torres P. Desfougères Y. Saiardi A. (2021). Inositol phosphate kinases in the eukaryote landscape. Adv. Biol. Regul. 79, 100782. 10.1016/j.jbior.2020.100782 33422459 PMC8024741

[B113] Laporte J. Bedez F. Bolino A. Mandel J. L. (2003). Myotubularins, a large disease-associated family of cooperating catalytically active and inactive phosphoinositides phosphatases. Hum. Mol. Genet. 12 (Suppl. l_2), R285–R292. 10.1093/hmg/ddg273 12925573

[B114] Laporte J. Hu L. J. Kretz C. Mandel J. L. Kioschis P. Coy J. F. (1996). A gene mutated in X–linked myotubular myopathy defines a new putative tyrosine phosphatase family conserved in yeast. Nat. Genet. 13 (2), 175–182. 10.1038/ng0696-175 8640223

[B115] Lasonder E. Green J. L. Grainger M. Langsley G. Holder A. A. (2015). Extensive differential protein phosphorylation as intraerythrocytic Plasmodium falciparum schizonts develop into extracellular invasive merozoites. Proteomics 15 (15), 2716–2729. 10.1002/pmic.201400508 25886026

[B116] Lazarus M. D. Schneider T. G. Taraschi T. F. (2008). A new model for hemoglobin ingestion and transport by the human malaria parasite Plasmodium falciparum. J. Cell Sci. 121 (11), 1937–1949. 10.1242/jcs.023150 18477610 PMC5105679

[B117] Leber W. Skippen A. Fivelman Q. L. Bowyer P. W. Cockcroft S. Baker D. A. (2009). A unique phosphatidylinositol 4-phosphate 5-kinase is activated by ADP-ribosylation factor in Plasmodium falciparum. Int. J. Parasitol. 39 (6), 645–653. 10.1016/j.ijpara.2008.11.015 19171150

[B118] Lecompte O. Poch O. Laporte J. (2008). PtdIns5P regulation through evolution: roles in membrane trafficking? Trends Biochem. Sci. 33 (10), 453–460. 10.1016/j.tibs.2008.07.002 18774718

[B119] Leirião P. Albuquerque S. S. Corso S. Van Gemert G. J. Sauerwein R. W. Rodriguez A. (2005). HGF/MET signalling protects Plasmodium‐infected host cells from apoptosis. Cell. Microbiol. 7 (4), 603–609. 10.1111/j.1462-5822.2004.00490.x 15760460

[B120] Lemmon M. A. Ferguson K. M. O'Brien R. Sigler P. B. Schlessinger J. (1995). Specific and high-affinity binding of inositol phosphates to an isolated pleckstrin homology domain. Proc. Natl. Acad. Sci. 92 (23), 10472–10476. 10.1073/pnas.92.23.10472 7479822 PMC40633

[B121] Li X. Rivas M. P. Fang M. Marchena J. Mehrotra B. Chaudhary A. (2002). Analysis of oxysterol binding protein homologue Kes1p function in regulation of Sec14p-dependent protein transport from the yeast Golgi complex. J. Cell Biol. 157 (1), 63–77. 10.1083/jcb.200201037 11916983 PMC2173257

[B122] Liendo A. Stedman T. T. Ngô H. M. Chaturvedi S. Hoppe H. C. Joiner K. A. (2001). Toxoplasma gondii ADP-ribosylation factor 1 mediates enhanced release of constitutively secreted dense granule proteins. J. Biol. Chem. 276 (21), 18272–18281. 10.1074/jbc.M008352200 11278405

[B123] Liu Y. Bankaitis V. A. (2010). Phosphoinositide phosphatases in cell biology and disease. Prog. lipid Res. 49 (3), 201–217. 10.1016/j.plipres.2009.12.001 20043944 PMC2873057

[B124] Lourido S. Tang K. Sibley L. D. (2012). Distinct signalling pathways control Toxoplasma egress and host‐cell invasion. EMBO J. 31 (24), 4524–4534. 10.1038/emboj.2012.299 23149386 PMC3545288

[B125] Marat A. L. Haucke V. (2016). Phosphatidylinositol 3‐phosphates—At the interface between cell signalling and membrane traffic. EMBO J. 35 (6), 561–579. 10.15252/embj.201593564 26888746 PMC4801949

[B126] Martorelli Di Genova B. Knoll L. J. (2020). Comparisons of the sexual cycles for the coccidian parasites Eimeria and Toxoplasma. Front. Cell. Infect. Microbiol. 10, 604897. 10.3389/fcimb.2020.604897 33381466 PMC7768002

[B127] Mbengue A. Bhattacharjee S. Pandharkar T. Liu H. Estiu G. Stahelin R. V. (2015). A molecular mechanism of artemisinin resistance in Plasmodium falciparum malaria. Nature 520 (7549), 683–687. 10.1038/nature14412 25874676 PMC4417027

[B128] McFadden G. I. (2000). Mergers and acquisitions: malaria and the great chloroplast heist. Genome Biol. 1 (4), REVIEWS1026–4. 10.1186/gb-2000-1-4-reviews1026 11178253 PMC138874

[B129] McFadden G. I. (2011). The apicoplast. Protoplasma 248 (4), 641–650. 10.1007/s00709-010-0250-5 21165662

[B130] McGovern O. L. Rivera‐Cuevas Y. Kannan G. Narwold A. J. Jr Carruthers V. B. (2018). Intersection of endocytic and exocytic systems in Toxoplasma gondii. Traffic 19 (5), 336–353. 10.1111/tra.12556 29437275 PMC6787929

[B131] McNamara C. W. Lee M. Lim C. S. Lim S. H. Roland J. Nagle A. (2013). Targeting Plasmodium PI(4)K to eliminate malaria. Nature 504 (7479), 248–253. 10.1038/nature12782 24284631 PMC3940870

[B132] Milani K. J. Schneider T. G. Taraschi T. F. (2015). Defining the morphology and mechanism of the hemoglobin transport pathway in Plasmodium falciparum-infected erythrocytes. Eukaryot. Cell 14 (4), 415–426. 10.1128/EC.00267-14 25724884 PMC4385801

[B225] Michell R. H. (2008). Inositol derivatives: evolution and functions. Nat. Rev. Mol. Cell Biol. 9 (2), 151–161. 18216771 10.1038/nrm2334

[B133] Mitra P. Zhang Y. Rameh L. E. Ivshina M. P. McCollum D. Nunnari J. J. (2004). A novel phosphatidylinositol (3, 4, 5) P3 pathway in fission yeast. J. Cell Biol. 166 (2), 205–211. 10.1083/jcb.200404150 15249580 PMC2172303

[B134] Mizushima N. Levine B. Cuervo A. M. Klionsky D. J. (2008). Autophagy fights disease through cellular self-digestion. Nature 451 (7182), 1069–1075. 10.1038/nature06639 18305538 PMC2670399

[B135] Morlon‐Guyot J. Berry L. Chen C. T. Gubbels M. J. Lebrun M. Daher W. (2014). The Toxoplasma gondii calcium‐dependent protein kinase 7 is involved in early steps of parasite division and is crucial for parasite survival. Cell. Microbiol. 16 (1), 95–114. 10.1111/cmi.12186 24011186 PMC4091637

[B136] Mousley C. J. Yuan P. Gaur N. A. Trettin K. D. Nile A. H. Deminoff S. J. (2012). A sterol-binding protein integrates endosomal lipid metabolism with TOR signaling and nitrogen sensing. Cell 148 (4), 702–715. 10.1016/j.cell.2011.12.026 22341443 PMC3285437

[B137] Muniz-Feliciano L. Van Grol J. Portillo J. A. C. Liew L. Liu B. Carlin C. R. (2013). Toxoplasma gondii-induced activation of EGFR prevents autophagy protein-mediated killing of the parasite. PLoS Pathog. 9 (12), e1003809. 10.1371/journal.ppat.1003809 24367261 PMC3868508

[B138] Nakatsu F. Baskin J. M. Chung J. Tanner L. B. Shui G. Lee S. Y. (2012). PtdIns4P synthesis by PI4KIIIα at the plasma membrane and its impact on plasma membrane identity. J. Cell Biol. 199 (6), 1003–1016. 10.1083/jcb.201206095 23229899 PMC3518224

[B139] Nicot A. S. Fares H. Payrastre B. Chisholm A. D. Labouesse M. Laporte J. (2006). The phosphoinositide kinase PIKfyve/Fab1p regulates terminal lysosome maturation in *Caenorhabditis elegans* . Mol. Biol. Cell 17 (7), 3062–3074. 10.1091/mbc.e05-12-1120 16801682 PMC1483040

[B140] Niebuhr K. Giuriato S. Pedron T. Philpott D. J. Gaits F. Sable J. (2002). Conversion of PtdIns (4, 5) P2 into PtdIns (5) P by the S. flexneri effector IpgD reorganizes host cell morphology. EMBO J. 21 (19), 5069–5078. 10.1093/emboj/cdf522 12356723 PMC129044

[B141] Nile A. H. Tripathi A. Yuan P. Mousley C. J. Suresh S. Wallace I. M. (2014). PITPs as targets for selectively interfering with phosphoinositide signaling in cells. Nat. Chem. Biol. 10 (1), 76–84. 10.1038/nchembio.1389 24292071 PMC4059020

[B142] Nilsson S. K. Childs L. M. Buckee C. Marti M. (2015). Targeting human transmission biology for malaria elimination. PLoS Pathog. 11 (6), e1004871. 10.1371/journal.ppat.1004871 26086192 PMC4472755

[B143] Nofal S. D. Dominicus C. Broncel M. Katris N. J. Flynn H. R. Arrizabalaga G. (2022). A positive feedback loop mediates crosstalk between calcium, cyclic nucleotide and lipid signalling in calcium-induced Toxoplasma gondii egress. PLoS Pathog. 18 (10), e1010901. 10.1371/journal.ppat.1010901 36265000 PMC9624417

[B144] O'Neal A. J. Butler L. R. Rolandelli A. Gilk S. D. Pedra J. H. (2020). Lipid hijacking: a unifying theme in vector-borne diseases. Elife 9, e61675. 10.7554/eLife.61675 33118933 PMC7595734

[B145] Oborník M. (2020). Photoparasitism as an intermediate state in the evolution of apicomplexan parasites. Trends Parasitol. 36 (9), 727–734. 10.1016/j.pt.2020.06.002 32680786

[B146] Ono H. Katagiri H. Funaki M. Anai M. Inukai K. Fukushima Y. (2001). Regulation of phosphoinositide metabolism, Akt phosphorylation, and glucose transport by PTEN (phosphatase and tensin homolog deleted on chromosome 10) in 3T3-L1 adipocytes. Mol. Endocrinol. 15 (8), 1411–1422. 10.1210/mend.15.8.0684 11463863

[B147] Parussini F. Coppens I. Shah P. P. Diamond S. L. Carruthers V. B. (2010). Cathepsin L occupies a vacuolar compartment and is a protein maturase within the endo/exocytic system of Toxoplasma gondii. Mol. Microbiol. 76 (6), 1340–1357. 10.1111/j.1365-2958.2010.07181.x 20444089 PMC2909120

[B148] Pathak S. Gauba R. Dantu S. C. Sheth D. Kale A. (2019). “Formin: the multidomain elongator of actin polymer,” in Actin polymerization in apicomplexan (Singapore: Springer), 29–38.

[B149] Petiot A. Fauré J. Stenmark H. Gruenberg J. (2003). PI3P signaling regulates receptor sorting but not transport in the endosomal pathway. J. Cell Biol. 162 (6), 971–979. 10.1083/jcb.200303018 12975344 PMC2172844

[B150] Piao H. Mayinger P. (2012). Growth and metabolic control of lipid signalling at the Golgi. Biochem. Soc. Trans. 40 (1), 205–209. 10.1042/BST20110637 22260691

[B151] Polevoy G. Wei H. C. Wong R. Szentpetery Z. Kim Y. J. Goldbach P. (2009). Dual roles for the Drosophila PI 4-kinase four wheel drive in localizing Rab11 during cytokinesis. J. Cell Biol. 187 (6), 847–858. 10.1083/jcb.200908107 19995935 PMC2806325

[B152] Qi Y. Y. Zhou X. J. Cheng F. J. Hou P. Ren Y. L. Wang S. X. (2018). Increased autophagy is cytoprotective against podocyte injury induced by antibody and interferon-α in lupus nephritis. Ann. rheumatic Dis. 77 (12), 1799–1809. 10.1136/annrheumdis-2018-213028 PMC6800572 30209031

[B153] Raabe A. Berry L. Sollelis L. Cerdan R. Tawk L. Vial H. J. (2011a). Genetic and transcriptional analysis of phosphoinositide-specific phospholipase C in Plasmodium. Exp. Parasitol. 129 (1), 75–80. 10.1016/j.exppara.2011.05.023 21651909

[B154] Raabe A. C. Wengelnik K. Billker O. Vial H. J. (2011b). Multiple roles for Plasmodium berghei phosphoinositide‐specific phospholipase C in regulating gametocyte activation and differentiation. Cell. Microbiol. 13 (7), 955–966. 10.1111/j.1462-5822.2011.01591.x 21518218 PMC3132445

[B155] Rameh L. (2010). “Type 2 PI4P-kinases,” in Handbook of cell signaling. Editors Bradshaw R. A. Dennis E. A. (Cambridge: Elsevier Academic Press), p1043–p1048.

[B156] Read M. Sherwin T. Holloway S. P. Gull K. Hyde J. E. (1993). Microtubular organization visualized by immunofluorescence microscopy during erythrocytic schizogony in Plasmodium falciparum and investigation of post-translational modifications of parasite tubulin. Parasitology 106 (3), 223–232. 10.1017/s0031182000075041 8488059

[B157] Ren B. Kong P. Hedar F. Brouwers J. F. Gupta N. (2020). Phosphatidylinositol synthesis, its selective salvage, and inter-regulation of anionic phospholipids in Toxoplasma gondii. Commun. Biol. 3 (1), 1–15. 10.1038/s42003-020-01480-5 33303967 PMC7728818

[B158] Ren J. Schaaf G. Bankaitis V. A. Ortlund E. A. Pathak M. C. (2011). Crystallization and preliminary X-ray diffraction analysis of Sfh3, a member of the Sec14 protein superfamily. Acta Crystallogr. Sect. F Struct. Biol. Cryst. Commun. 67 (10), 1239–1243. 10.1107/S1744309111027096 PMC3212372 22102037

[B159] Rivas M. P. Kearns B. G. Xie Z. Guo S. Sekar M. C. Hosaka K. (1999). Pleiotropic alterations in lipid metabolism in yeast sac1 mutants: relationship to “bypass Sec14p” and inositol auxotrophy. Mol. Biol. Cell 10 (7), 2235–2250. 10.1091/mbc.10.7.2235 10397762 PMC25439

[B160] Roiko M. S. Carruthers V. B. (2013). Functional dissection of Toxoplasma gondii perforin-like protein 1 reveals a dual domain mode of membrane binding for cytolysis and parasite egress. J. Biol. Chem. 288 (12), 8712–8725. 10.1074/jbc.M113.450932 23376275 PMC3605689

[B161] Rostislavleva K. Soler N. Ohashi Y. Zhang L. Pardon E. Burke J. E. (2015). Structure and flexibility of the endosomal Vps34 complex reveals the basis of its function on membranes. Science 350 (6257), aac7365. 10.1126/science.aac7365 26450213 PMC4601532

[B162] Routt S. M. Ryan M. M. Tyeryar K. Rizzieri K. E. Mousley C. Roumanie O. (2005). Nonclassical PITPs activate PLD via the Stt4p PtdIns‐4‐kinase and modulate function of late stages of exocytosis in vegetative yeast. Traffic 6 (12), 1157–1172. 10.1111/j.1600-0854.2005.00350.x 16262726

[B163] Sahu S. Wang Z. Jiao X. Gu C. Jork N. Wittwer C. (2020). InsP7 is a small-molecule regulator of NUDT3-mediated mRNA decapping and processing-body dynamics. Proc. Natl. Acad. Sci. 117 (32), 19245–19253. 10.1073/pnas.1922284117 32727897 PMC7431097

[B164] Sbrissa D. Ikonomov O. C. Filios C. Delvecchio K. Shisheva A. (2012). Functional dissociation between PIKfyve-synthesized PtdIns5P and PtdIns (3, 5) P2 by means of the PIKfyve inhibitor YM201636. Am. J. Physiology-Cell Physiology 303 (4), C436–C446. 10.1152/ajpcell.00105.2012 PMC3422984 22621786

[B165] Schaaf G. Ortlund E. A. Tyeryar K. R. Mousley C. J. Ile K. E. Garrett T. A. (2008). Functional anatomy of phospholipid binding and regulation of phosphoinositide homeostasis by proteins of the sec14 superfamily. Mol. Cell 29 (2), 191–206. 10.1016/j.molcel.2007.11.026 18243114 PMC7808562

[B166] Schorr M. Then A. Tahirovic S. Hug N. Mayinger P. (2001). The phosphoinositide phosphatase Sac1p controls trafficking of the yeast Chs3p chitin synthase. Curr. Biol. 11 (18), 1421–1426. 10.1016/s0960-9822(01)00449-3 11566100

[B167] Sechi S. Colotti G. Belloni G. Mattei V. Frappaolo A. Raffa G. D. (2014). GOLPH3 is essential for contractile ring formation and Rab11 localization to the cleavage site during cytokinesis in *Drosophila melanogaster* . PLoS Genet. 10 (5), e1004305. 10.1371/journal.pgen.1004305 24786584 PMC4006750

[B168] Séron K. Dzierszinski F. Tomavo S. (2000). Molecular cloning, functional complementation in *Saccharomyces cerevisiae* and enzymatic properties of phosphatidylinositol synthase from the protozoan parasite Toxoplasma gondii. Eur. J. Biochem. 267 (22), 6571–6579. 10.1046/j.1432-1327.2000.01749.x 11054108

[B169] Sha B. Phillips S. E. Bankaitis V. A. Luo M. (1998). Crystal structure of the *Saccharomyces cerevisiae* phosphatidylinositol-transfer protein. Nature 391 (6666), 506–510. 10.1038/35179 9461221

[B170] Shin H. W. Hayashi M. Christoforidis S. Lacas-Gervais S. Hoepfner S. Wenk M. R. (2005). An enzymatic cascade of Rab5 effectors regulates phosphoinositide turnover in the endocytic pathway. J. Cell Biol. 170 (4), 607–618. 10.1083/jcb.200505128 16103228 PMC2171494

[B171] Shisheva A. (2008). PIKfyve: partners, significance, debates and paradoxes. Cell Biol. Int. 32 (6), 591–604. 10.1016/j.cellbi.2008.01.006 18304842 PMC2491398

[B172] Shisheva A. (2013). PtdIns5P: news and views of its appearance, disappearance and deeds. Archives Biochem. biophysics 538 (2), 171–180. 10.1016/j.abb.2013.07.023 PMC4475639 23916588

[B173] Sidik S. M. Huet D. Ganesan S. M. Huynh M. H. Wang T. Nasamu A. S. (2016). A genome-wide CRISPR screen in Toxoplasma identifies essential Apicomplexan genes. Cell 166 (6), 1423–1435.e12. 10.1016/j.cell.2016.08.019 27594426 PMC5017925

[B174] Sinai A. P. Roepe P. D. (2012). Autophagy in apicomplexa: a life sustaining death mechanism? Trends Parasitol. 28 (9), 358–364. 10.1016/j.pt.2012.06.006 22819059 PMC4354876

[B175] Singh S. Alam M. M. Pal-Bhowmick I. Brzostowski J. A. Chitnis C. E. (2010). Distinct external signals trigger sequential release of apical organelles during erythrocyte invasion by malaria parasites. PLoS Pathog. 6 (2), e1000746. 10.1371/journal.ppat.1000746 20140184 PMC2816683

[B176] So T. Croft M. (2007). Cutting edge: OX40 inhibits TGF-β-and antigen-driven conversion of naive CD4 T cells into CD25+ Foxp3+ T cells. J. Immunol. 179 (3), 1427–1430. 10.4049/jimmunol.179.3.1427 17641007

[B177] Stefan C. J. Audhya A. Emr S. D. (2002). The yeast synaptojanin-like proteins control the cellular distribution of phosphatidylinositol (4, 5)-bisphosphate. Mol. Biol. Cell 13 (2), 542–557. 10.1091/mbc.01-10-0476 11854411 PMC65648

[B178] Sternberg A. R. Roepe P. D. (2020). Heterologous expression, purification, and functional analysis of the Plasmodium falciparum phosphatidylinositol 4-kinase IIIβ. Biochemistry 59 (27), 2494–2506. 10.1021/acs.biochem.0c00259 32543181

[B179] Stortz J. F. Del Rosario M. Singer M. Wilkes J. M. Meissner M. Das S. (2019). Formin-2 drives polymerisation of actin filaments enabling segregation of apicoplasts and cytokinesis in Plasmodium falciparum. Elife 8, e49030. 31322501 10.7554/eLife.49030 PMC6688858

[B180] Strahl T. Thorner J. (2007). Synthesis and function of membrane phosphoinositides in budding yeast, *Saccharomyces cerevisiae* . Mol. Cell Biol. Lipids 1771 (3), 353–404. 10.1016/j.bbalip.2007.01.015 PMC1868553 17382260

[B181] Streb H. Irvine R. F. Berridge M. J. Schulz I. (1983). Release of Ca2+ from a nonmitochondrial intracellular store in pancreatic acinar cells by inositol-1, 4, 5-trisphosphate. Nature 306 (5938), 67–69. 10.1038/306067a0 6605482

[B182] Sudhakar R. Das D. Thanumalayan S. Gorde S. Sijwali P. S. (2021). Plasmodium falciparum Atg18 localizes to the food vacuole via interaction with the multi-drug resistance protein 1 and phosphatidylinositol 3-phosphate. Biochem. J. 478 (9), 1705–1732. 10.1042/BCJ20210001 33843972

[B183] Sugiura T. Takahashi C. Chuma Y. Fukuda M. Yamada M. Yoshida U. (2019). Biophysical parameters of the Sec14 phospholipid exchange cycle. Biophysical J. 116 (1), 92–103. 10.1016/j.bpj.2018.11.3131 PMC6342728 30580923

[B184] Sugiyama M. G. Fairn G. D. Antonescu C. N. (2019). Akt-ing up just about everywhere: compartment-specific Akt activation and function in receptor tyrosine kinase signaling. Front. Cell Dev. Biol. 7, 70. 10.3389/fcell.2019.00070 31131274 PMC6509475

[B185] Taku I. Hirai T. Makiuchi T. Shinzawa N. Iwanaga S. Annoura T. (2021). Rab5b-associated Arf1 GTPase regulates export of N-myristoylated adenylate kinase 2 from the endoplasmic reticulum in Plasmodium falciparum. Front. Cell. Infect. Microbiol. 10, 908. 10.3389/fcimb.2020.610200 PMC7884776 33604307

[B186] Tawk L. Chicanne G. Dubremetz J. F. Richard V. Payrastre B. Vial H. J. (2010). Phosphatidylinositol 3-phosphate, an essential lipid in Plasmodium, localizes to the food vacuole membrane and the apicoplast. Eukaryot. Cell 9 (10), 1519–1530. 10.1128/EC.00124-10 20709789 PMC2950420

[B187] Tawk L. Dubremetz J. F. Montcourrier P. Chicanne G. Merezegue F. Richard V. (2011). Phosphatidylinositol 3-monophosphate is involved in toxoplasma apicoplast biogenesis. PLoS Pathog. 7 (2), e1001286. 10.1371/journal.ppat.1001286 21379336 PMC3040667

[B188] Tengholm A. Idevall‐Hagren O. (2009). Spatio‐temporal dynamics of phosphatidylinositol‐3, 4, 5‐trisphosphate signalling. Vitamins Hormones 80, 287–311. 10.1016/S0083-6729(08)00611-0 19251042

[B189] Thériault C. Richard D. (2017). Characterization of a putative Plasmodium falciparum SAC1 phosphoinositide-phosphatase homologue potentially required for survival during the asexual erythrocytic stages. Sci. Rep. 7 (1), 12710–12719. 10.1038/s41598-017-12762-0 28983103 PMC5629215

[B226] Tomavo S. Slomianny C. Meissner M. Carruthers V. B. (2013). Protein trafficking through the endosomal system prepares intracellular parasites for a home invasion. PLoS pathogens 9 (10), e1003629. 24204248 10.1371/journal.ppat.1003629 PMC3812028

[B190] Toyoshima F. Matsumura S. Morimoto H. Mitsushima M. Nishida E. (2007). PtdIns(3,4,5)P_3_ regulates spindle orientation in adherent cells. Dev. Cell 13 (6), 796–811. 10.1016/j.devcel.2007.10.014 18061563

[B191] Vaid A. Ranjan R. Smythe W. A. Hoppe H. C. Sharma P. (2010). PfPI3K, a phosphatidylinositol-3 kinase from Plasmodium falciparum, is exported to the host erythrocyte and is involved in hemoglobin trafficking. Blood*, J. Am. Soc. Hematol.* 115 (12), 2500–2507. 10.1182/blood-2009-08-238972 PMC2918364 20093402

[B192] van Ooij C. Withers-Martinez C. Ringel A. Cockcroft S. Haldar K. Blackman M. J. (2013). Identification of a Plasmodium falciparum phospholipid transfer protein. J. Biol. Chem. 288 (44), 31971–31983. 10.1074/jbc.M113.474189 24043620 PMC3814793

[B193] Vanhaesebroeck B. Guillermet-Guibert J. Graupera M. Bilanges B. (2010). The emerging mechanisms of isoform-specific PI3K signalling. Nat. Rev. Mol. Cell Biol. 11 (5), 329–341. 10.1038/nrm2882 20379207

[B194] Venditti R. Masone M. C. Wilson C. De Matteis M. A. (2016). PI (4) P homeostasis: who controls the controllers? Adv. Biol. Regul. 60, 105–114. 10.1016/j.jbior.2015.09.007 26542744

[B195] Venugopal K. Chehade S. Werkmeister E. Barois N. Periz J. Lafont F. (2020a). Rab11A regulates dense granule transport and secretion during Toxoplasma gondii invasion of host cells and parasite replication. PLoS Pathog. 16 (5), e1008106. 10.1371/journal.ppat.1008106 32463830 PMC7255593

[B196] Venugopal K. Hentzschel F. Valkiūnas G. Marti M. (2020b). Plasmodium asexual growth and sexual development in the haematopoietic niche of the host. Nat. Rev. Microbiol. 18 (3), 177–189. 10.1038/s41579-019-0306-2 31919479 PMC7223625

[B197] Vergne I. Deretic V. (2010). The role of PI3P phosphatases in the regulation of autophagy. FEBS Lett. 584 (7), 1313–1318. 10.1016/j.febslet.2010.02.054 20188094 PMC2885894

[B198] Verstreken P. Koh T. W. Schulze K. L. Zhai R. G. Hiesinger P. R. Zhou Y. (2003). Synaptojanin is recruited by endophilin to promote synaptic vesicle uncoating. Neuron 40 (4), 733–748. 10.1016/s0896-6273(03)00644-5 14622578

[B199] Vial H. J. Eldin P. Tielens A. G. van Hellemond J. J. (2003). Phospholipids in parasitic protozoa. Mol. Biochem. Parasitol. 126 (2), 143–154. 10.1016/s0166-6851(02)00281-5 12615313

[B200] Vicinanza M. Korolchuk V. I. Ashkenazi A. Puri C. Menzies F. M. Clarke J. H. (2015). PI (5) *P* regulates autophagosome biogenesis. Mol. Cell 57 (2), 219–234. 10.1016/j.molcel.2014.12.007 25578879 PMC4306530

[B201] Vivanco I. Sawyers C. L. (2002). The phosphatidylinositol 3-kinase–AKT pathway in human cancer. Nat. Rev. Cancer 2 (7), 489–501. 10.1038/nrc839 12094235

[B202] Walch-Solimena C. Novick P. (1999). The yeast phosphatidylinositol-4-OH kinase pik1 regulates secretion at the Golgi. Nat. Cell Biol. 1 (8), 523–525. 10.1038/70319 10587649

[B203] Wang H. Sun H. Q. Zhu X. Zhang L. Albanesi J. Levine B. (2015). GABARAPs regulate PI4P-dependent autophagosome: lysosome fusion. Proc. Natl. Acad. Sci. 112 (22), 7015–7020. 10.1073/pnas.1507263112 26038556 PMC4460452

[B204] Wang S. Zhang Z. Wang Y. Gadahi J. A. Xie Q. Xu L. (2017). *Toxoplasma gondii* excretory/secretory antigens (TgESAs) suppress pro-inflammatory cytokine secretion by inhibiting TLR-induced NF-κB activation in LPS-stimulated murine macrophages. Oncotarget 8 (51), 88351–88359. 10.18632/oncotarget.19362 29179440 PMC5687610

[B205] Wang Y. Fang R. Yuan Y. Pan M. Hu M. Zhou Y. (2016). Identification of host proteins, Spata3 and Dkk2, interacting with Toxoplasma gondii micronemal protein MIC3. Parasitol. Res. 115 (7), 2825–2835. 10.1007/s00436-016-5033-2 27053129

[B206] Wei Y. J. Sun H. Q. Yamamoto M. Wlodarski P. Kunii K. Martinez M. (2002). Type II phosphatidylinositol 4-kinase β is a cytosolic and peripheral membrane protein that is recruited to the plasma membrane and activated by Rac-GTP. J. Biol. Chem. 277 (48), 46586–46593. 10.1074/jbc.M206860200 12324459

[B207] Welti R. Mui E. Sparks A. Wernimont S. Isaac G. Kirisits M. (2007). Lipidomic analysis of Toxoplasma gondii reveals unusual polar lipids. Biochemistry 46 (48), 13882–13890. 10.1021/bi7011993 17988103 PMC2576749

[B208] Wen Y. Vogt V. M. Feigenson G. W. (2021). PI(4,5)P2 clustering and its impact on biological functions. Annu. Rev. Biochem. 90, 681–707. 10.1146/annurev-biochem-070920-094827 33441034

[B209] Wengelnik K. Daher W. Lebrun M. (2018). Phosphoinositides and their functions in Apicomplexan parasites. Int. J. Parasitol. 48 (7), 493–504. 10.1016/j.ijpara.2018.01.009 29596862

[B210] Wengelnik K. Vial H. J. (2007). Characterisation of the phosphatidylinositol synthase gene of Plasmodium species. Res. Microbiol. 158 (1), 51–59. 10.1016/j.resmic.2006.11.005 17223316

[B211] Whitley P. Hinz S. Doughty J. (2009). Arabidopsis FAB1/PIKfyve proteins are essential for development of viable pollen. Plant physiol. 151 (4), 1812–1822. 10.1104/pp.109.146159 19846542 PMC2785992

[B212] Whitters E. A. Cleves A. E. McGee T. P. Skinner H. B. Bankaitis V. A. (1993). SAC1p is an integral membrane protein that influences the cellular requirement for phospholipid transfer protein function and inositol in yeast. J. Cell Biol. 122 (1), 79–94. 10.1083/jcb.122.1.79 8314848 PMC2119615

[B213] Wiersma H. I. Galuska S. E. Tomley F. M. Sibley L. D. Liberator P. A. Donald R. G. (2004). A role for coccidian cGMP-dependent protein kinase in motility and invasion. Int. J. Parasitol. 34 (3), 369–380. 10.1016/j.ijpara.2003.11.019 15003497

[B214] Wood C. S. Hung C. S. Huoh Y. S. Mousley C. J. Stefan C. J. Bankaitis V. (2012). Local control of phosphatidylinositol 4-phosphate signaling in the Golgi apparatus by Vps74 and Sac1 phosphoinositide phosphatase. Mol. Biol. Cell 23 (13), 2527–2536. 10.1091/mbc.E12-01-0077 22553352 PMC3386216

[B215] Wu W. I. Routt S. Bankaitis V. A. Voelker D. R. (2000). A new gene involved in the transport-dependent metabolism of phosphatidylserine, PSTB2/PDR17, shares sequence similarity with the gene encoding the phosphatidylinositol/phosphatidylcholine transfer protein, SEC14. J. Biol. Chem. 275 (19), 14446–14456. 10.1074/jbc.275.19.14446 10799527

[B216] Xie Z. Hur S. K. Zhao L. Abrams C. S. Bankaitis V. A. (2018). A Golgi lipid signaling pathway controls apical Golgi distribution and cell polarity during neurogenesis. Dev. Cell 44 (6), 725–740.e4. 10.1016/j.devcel.2018.02.025 29587143 PMC5877119

[B217] Yang T. Yeoh L. M. Tutor M. V. Dixon M. W. McMillan P. J. Xie S. C. (2019). Decreased K13 abundance reduces hemoglobin catabolism and proteotoxic stress, underpinning artemisinin resistance. Cell Rep. 29 (9), 2917–2928.e5. 10.1016/j.celrep.2019.10.095 31775055

[B218] Yoder M. D. Thomas L. M. Tremblay J. M. Oliver R. L. Yarbrough L. R. Helmkamp G. M. (2001). Structure of a multifunctional protein: mammalian phosphatidylinositol transfer protein complexed with phosphatidylcholine. J. Biol. Chem. 276 (12), 9246–9252. 10.1074/jbc.M010131200 11104777

[B219] Zeng B. Cai X. Zhu G. (2006). Functional characterization of a fatty acyl-CoA binding protein (ACBP) from the Apicomplexan Cryptosporidium parvum. Microbiol. Read. Engl. 152 (Pt 8), 2355–2363. 10.1099/mic.0.28944-0 PMC1513434 16849800

[B220] Zhang X. Loijens J. C. Boronenkov I. V. Parker G. J. Norris F. A. Chen J. (1997). Phosphatidylinositol-4-phosphate 5-kinase isozymes catalyze the synthesis of 3-phosphate-containing phosphatidylinositol signaling molecules. J. Biol. Chem. 272, 17756–17761. 10.1074/jbc.272.28.17756 9211928

[B221] Zhao X. Várnai P. Tuymetova G. Balla A. Tóth Z. E. Oker-Blom C. (2001). Interaction of neuronal calcium sensor-1 (NCS-1) with phosphatidylinositol 4-kinase β stimulates lipid kinase activity and affects membrane trafficking in COS-7 cells. J. Biol. Chem. 276 (43), 40183–40189. 10.1074/jbc.M104048200 11526106

[B222] Zhu W. Li J. Pappoe F. Shen J. Yu L. (2019). Strategies developed by toxoplasma gondii to survive in the host. Front. Microbiol. 10, 899. 10.3389/fmicb.2019.00899 31080445 PMC6497798

[B224] Zolov S. N. Bridges D. Zhang Y. Lee W. W. Riehle E. Verma R. (2012). *In vivo*, Pikfyve generates PI(3,5)P2, which serves as both a signaling lipid and the major precursor for PI5P. Proc. Natl. Acad. Sci. 109 (43), 17472–17477. 10.1073/pnas.1203106109 23047693 PMC3491506

