# Supplementary material for: Regulation of phosphoinositide metabolism in Apicomplexan parasites
Source: Front Cell Dev Biol. 2023 Sep 15;11:1163574. doi: 10.3389/fcell.2023.1163574 (PMC10543664; doi:10.3389/fcell.2023.1163574)
Supplement: Supplementary file 1 [file Table1.pdf]

Supplementary Table 1. Summary of PI, PIP kinases, PIP phosphatases and accessory protein homologues in *Plasmodium* spp. and *T. gondii*. (*) indicates putative orthologues found on the PlasmoDB with e-value < 10^{-5}. (nf): not orthologue was found with e-value < 10^{-5}. (nd): genes which phenotypic score was not determined in *P. berghei* and/or in *T. gondii* in their respective studies of wide genome screen for identification of essential genes (Bushell et al., 2016 and Sidik et al., 2016).

Supplementary Table 1.

PIP	Protein	*P.falciparum*	*P.berghei*	Growth rate phenotypes in *P. berghei*	*T. gondii*	Phenotypic score in *T. gondii*	Function in Apicomplexan	Reference
**PtdIns3P**	Class III PI3K/Vps34	PF3D7_0515300 (PfPI3KIII)	PBANKA_111490 (PbPI3KIII)	Essential	TGME49_215700 (TgPI3KIII)	-3.88	Mitophagy and transport of Hb-loaded vesicles to the food vacuole (*Plasmodium* spp.). Transport of proteins to the apicoplast (*T. gondii*)	Tawk et al., 2010, 2011; Vaid et al., 2010; Daher et al., 2015
	Vps15	PF3D7_0823000*	PBANKA_010760*	nd	TGME49_310190*	-2.28		Besteiro et al., 2017
	Beclin-1/Vps30	nf	nf		TGME49_221360*	0.41		Besteiro et al., 2017
	ATG14L	nf	nf		nf			
	UVRAG/Vps38	nf	nf		nf			
	Kelch13	PF3D7_1343700 (PfKelch13)	PBANKA_1356700*	nd	nf		PfPI3K proteolysis	Mbengue et al., 2015
	AKT	PF3D7_1246900 (PfAKT)	PBANKA_1460000*	nd	TGME49_267540*, TGME49_286470*, TGME49_226030*	-0.78, -1.74 -3.04	PtdIns3P synthesis upregulation in *Plasmodium* spp.	Mbengue et al., 2015
	MTM	nf	nf		nf			
**PtdIns(3,5)P_2**	PIKfyve/Fab1	PF3D7_1412400 (PfPIKfyve)	PBANKA_103020 (PbPIKfyve)	Essential	TGME49_256960 & TGME49_256920 (TgPIKfyve)	-3.33 -4.83	Apicoplast biogenesis/maintenance	Daher et al., 2015

	Sac3/Fig4	PF3D7_0802500*	PBANKA_1227700*	Significantly slow	TGME49_256830*, TGME49_238400*, TGME49_316230*,	-4.73 -3.70 -1.35		
	ArPIKfyve/Vac14	PF3D7_1225700*	PBANKA_1440600*	Essential	TGME49_244040 (TgArPIKfyve)	-4.42	Apicoplast biogenesis/maintenance	Daher et al., 2015
	Vac7	nf	nf		nf			
	Atg18	PF3D7_1012900 (PfAtg18)	PBANKA_1211300*	Essential	TGME49_220160 (TgAtg18), TGME49_288600*	-1.46 2.23	Apicoplast biogenesis/maintenance	Bansal et al., 2017
**PtdIns(3,4,5)P$_3$**	Class I PI3K	nf	nf		nf			Tawk et al., 2010
	PTEN	PF3D7_1219000 (FRM2)	PBANKA_1434600*	nd	TGME49_206580 (FRM2)	-1.12	Apicoplast maintenance	Pathak et al., 2019; Stortz et al., 2018
	SKIP (INPP5K)	PF3D7_0705500*	PBANKA_0803200*	Essential	TGME49_238400*, TGME49_288800*, TGME49_293190*	-3.70 0.51 1.18		
	SHIP1/2 (INPP5D/INPPL1)	PF3D7_0705500*	PBANKA_0803200*	Essential	TGME49_238400*, TGME49_293190*	-3.70 1.18		
	PIPP (INPP5J)	PF3D7_0705500*	PBANKA_0803200*	Essential	TGME49_238400*	-3.70		
**PtdIns(3,4)P$_2$**	Class II PI3K	nf	nf		nf			
	INPP4A	nf	nf		nf			
	INPP4B	nf	nf		nf			
**PtdIns4P**	PI4KIIα, PI4KIIβ/Lsb6	PF3D7_0311300*	PBANKA_0409300*	Significantly slow	TGME49_276170*	-3.91		
	PI4KIIIα/Stt4	PF3D7_0419900*	PBANKA_0722000*	Significantly slow	TGME49_228690*	-3.01		
	TTC7/Ypp1	nf	nf		nf			
	EFR3/Efr3	nf	nf		nf			

	PI4KIIIβ/Pik1	PF3D7_0509800 (PfPI4KIIIβ)	PBANKA_1109400 (PbPI4KIIIβ)	Essential	TGME49_296010 (TgPI4KIIIβ)	-3.60	Vesicle trafficking events.	McNamara et al., 2013; Stenberg & Roepe, 2020
	Arf1	PF3D7_1020900 (PfArf1)	PBANKA_0505100 (PbArf1)	Essential	TGME49_276140 (TgArf1), TGME49_262860*, TGME49_212950*, TGME49_215060*	-1.44 -0.52 -4.24 -6.29	Trafficking of PEXEL-positive and -negative proteins from ER. Stimulate secretion of preformed dense granule	Taku et al., 2021; Liendo et al., 2001; Venupogal et al., 2020; Leber et al., 2009
	NCS-1/Frq1	PF3D7_1451700*	PBANKA_1315400*	Significantly slow	TGME49_213800*, TGME49_230490*, TGME49_228750*, TGMAS_242400*, TGMAS_301440*	-3.62 -5.81 -4.13 1.30 -3.30		
	GGA2	nf	nf		TGME49_313670*	-4.27		
	PKD1/2	PF3D7_1123100 (CDPK7)	PBANKA_0925200 (CDPK7), PBANKA_1351500 (CDPK5)	Essential, Essential	TGME49_228750 (CDPK7), TGME49_224950 (CDPK7), TGME49_206590 (CDPK2A), TGME49_233905*	-4.13 1.69 -2.05 -3.54	Intraerythrocytic development and protein trafficking (Plasmodium spp.).	Brochet et al., 2014; Bansal et al., 2021
	14-3-3γ	PF3D7_0818200*	PBANKA_0712600*	Essential	TGME49_263090*, TGME49_269582*, TGME49_269960*	-5.79 0.62 -1.12		
	PKG1/2	PF3D7_1436600 (PfPKG)	PBANKA_1008200 (PbPKG)	nd	TGME49_311360 (TgPKG), TGME49_228420*, TGME49_286470*	-2.15 0.14, -1.74	Ookinete gliding and motility. Schizont rupture (Plasmodium spp.)	
	Sec14-like	PF3D7_0626400*, PF3D7_0629900*, PF3D7_1127600*	PBANKA_0614800*	nd	TGME49_254390*, TGME49_203390*, TGME49_246330*, TGME49_269390*	-2.16 -0.37 -0.23 0.09		

	START-like	PF3D7_1351000*<br>PF3D7_0104200	PBANKA_1363800*<br>PBANKA_0208900	nd<br>Essential	TGME49_289570*,	-1.70	Transfer of PIPs	Van Ooij et al., 2013; Hill et al., 2016
	Sac1	PF3D7_1354200<br>(PfSac1)	PBANKA_1130800*	nd	TGME49_316230*,<br>TGME49_238400*,<br>TGME49_256830*	-5.27<br>-3.70<br>-4.73	Regulation of PtdIns4P levels in ER and Golgi.	Thériault et al., 2017.
	OSBP/ORP	PF3D7_1131800*	PBANKA_0916600*	Significantly slow	TGME49_264760*,<br>TGME49_294320*,<br>TGME49_289570*	-6.07<br>0.88<br>-1.70		
	PI3K/PI4K-like mTOR	PF3D7_0515300*	nf		TGME49_316430*,<br>TGME49_283702*,<br>TGME49_248540*<br>&<br>TGME49_248530*	0.21<br>-2.68<br>-0.88<br><br>-2.17		
	Other PI3K/PI4K-like proteins	nf	PBANKA_1114900*	Essential	TGME49_266010*,<br>TGME49_268370*	-3.21<br>-3.56		
**PtdIns(4,5)P_2**	Type I PI4P5K/Mss4p	PF3D7_0110600<br>(PfPI4P5K)	PBANKA_0203100<br>(PbPI4P5K)	Essential	TGME49_230490<br>(TgPI4P5KA),<br>TGME49_245730<br>(TgPI4P5KB)	-5.81<br><br>-1.93	Modulation of PtdIns(4,5)P2 production in response to changes in intracellular calcium concentrations within the parasite.	Leber et al., 2009; Brochet et al., 2014
	Type II PI5P4K	nf	nf		nf			
	Arf1	PF3D7_1020900<br>(PfArf1)	PBANKA_0505100<br>(PbArf1)	Essential	TGME49_276140<br>(TgArf1),<br>TGME49_262860*,<br>TGME49_212950*,<br>TGME49_215060*	-1.44<br><br>-0.52<br>-4.24<br>-6.29	ER and trafficking of PEXEL-positive and -negative proteins, & stimulated secretion of preformed dense granule.	Taku et al., 2021; Liendo et al., 2001; Venupogal et al., 2020; Leber et al., 2009
	PKG1/2	PF3D7_1436600<br>(PfPKG)	PBANKA_1008200<br>(PbPKG)	nd	TGME49_311360<br>(TgPKG),<br>TGME49_228420*,<br>TGME49_286470*	-2.15<br><br>0.14<br>-1.74	Ookinete gliding motility and schizont rupture.	Brochet et al., 2014; Collins et al., 2013

	PLCδ	PF3D7_1013500 (PfPI-PLC)	PBANKA_121190 (PbPI-PLC)	Essential	TGME49_248830 (TgPI-PLC)	-4.54	Exocytosis of microneme proteins.	Raabe et al., 2011a, Raabe et al., 2011b, Bullen et al., 2016, Fang et al., 2006
	Synaptojanin1/2	PF3D7_1354200* (1), PF3D7_0705500* (2)	PBANKA_1130800* (1), PBANKA_0803200* (2)	nd Essential	TGME49_238400* (1/2), TGME49_316230* (1/2), TGME49_288800* (2)	-3.70 -1.35 0.51		
**PtdIns5P**	Vps15	PF3D7_1431500*, PF3D7_0623800*	nf		TGME49_310190*, TGME49_233010*, TGME49_233905*, TGME49_267540*	-2.28 -1.53 -3.54 -0.78		
	Class III PI3K	PF3D7_0515300	PBANKA_111490	Essential	TGME49_215700	-3.88		
	aMTM	nf	nf		nf			
	ArPIKfyve/Vac14	PF3D7_1225700*	PBANKA_1440600*	Essential	TGME49_244040 (TgArPIKfyve)	-4.42	Apicoplast biogenesis/maintenance	Daher et al., 2015
	PIKfyve/Fab1	PF3D7_1412400 (PfPIKfyve)	PBANKA_103020 (PbPIKfyve)	Essential	TGME49_256960 & TGME49_256920 (TgPIKfyve)	-3.33 -4.83	Apicoplast biogenesis/maintenance	Daher et al., 2015
	Fig4	PF3D7_0802500*	PBANKA_1227700*	Significantly slow	TGME49_256830*, TGME49_238400*, TGME49_316230*	-4.73 -3.70 -1.35		
	Class I PI3K	nf	nf		nf			
	dMTM	nf	nf		nf			
	aMTM	nf	nf		nf			
	Type II PI5P4K	nf	nf		nf			
	PtdIns(4,5)P2 4-phosphatases	nf	nf		nf			
